# Supplementary material for: Reef-scale trends in Florida Acropora spp. abundance and the effects of population enhancement
Source: PeerJ. 2016 Sep 29;4:e2523. doi: 10.7717/peerj.2523 (PMC5047146; doi:10.7717/peerj.2523)
Supplement: Table S1 — Total numbers of outplants for Acropora cervicornis (Ac) and A. palmata (Ap) by site and year as reported by the Coral Restoration Foundation (J. Levy and K. Ripple, pers. comm). [file peerj-04-2523-s002.docx]

Suppl. Table 1: Total numbers of outplants for *Acropora cervicornis* (Ac) and *A. palmata* (Ap) by site and year as reported by the Coral Restoration Foundation (J. Levy and K. Ripple, pers. comm).

|  | # Ac | | | | | | | | | # Ap | | | |
| --- | --- | --- | --- | --- | --- | --- | --- | --- | --- | --- | --- | --- | --- |
| Reef | 2007 | 2008 | 2009 | 2010 | 2011 | 2012 | 2013 | 2014 | 2015 | 2012 | 2013 | 2014 | 2015 |
| Carysfort | 0 | 0 | 0 | 0 | 0 | 0 | 0 | 370 | 815 | 0 | 0 | 60 | 6 |
| French Reef | 0 | 0 | 18 | 24 | 0 | 0 | 640 | 0 | 0 | 0 | 0 | 110 | 120 |
| Grecian Rocks | 0 | 0 | 0 | 0 | 0 | 0 | 0 | 300 | 605 | 0 | 0 | 0 | 3 |
| Molasses Reef | 18 | 18 | 86 | 0 | 575 | 789 | 1059 | 520 | 915 | 27 | 0 | 180 | 170 |
| North Dry Rocks | 0 | 0 | 0 | 0 | 0 | 0 | 0 | 300 | 380 | 0 | 0 | 50 | 120 |
| Pickles Reef | 0 | 18 | 24 | 0 | 0 | 515 | 1256 | 1690 | 3455 | 0 | 15 | 130 | 543 |
| White Bank Dry Rocks #2 | 0 | 18 | 24 | 0 | 0 | 0 | 665 | 600 | 0 | 0 | 0 | 0 | 0 |
