# Supplementary material for: Reef-scale trends in Florida Acropora spp. abundance and the effects of population enhancement
Source: PeerJ. 2016 Sep 29;4:e2523. doi: 10.7717/peerj.2523 (PMC5047146; doi:10.7717/peerj.2523)
Supplement: Table S2 — Names, abbreviations, coordinates, and census dates for each site arranged in alphabetical order. [file peerj-04-2523-s003.docx]

| Reef name | Abbreviation | Coordinates | Years surveyed |
| --- | --- | --- | --- |
| Admirals | AD | 25.0701°N, 80.3701° W | 2006, 2015 |
| Belgium | BE | 25.0418°N, 80.3435°W | 2005, 2015 |
| Cannon Patch | CP | 25.1089°N, 80.3384°W | 2007, 2015 |
| Carysfort | CF | 25.2227°N, 80.2105°W | 2005, 2014, 2015 |
| French | FR | 25.0342°N, 80.3490°W | 2007, 2014, 2015 |
| Grecian Rocks | GR | 25.1119°N, 80.3030°W | 2006, 2014, 2015 |
| Horseshoe (thicket only) | HS | 25.1401°N, 80.2949°W | 2005, 2007, 2014, 2015 |
| Little Grecian | LG | 25.1199°N, 80.3006°W | 2006, 2013, 2015 |
| Molasses | ML | 25.0100°N, 80.3741°W | 2006, 2014, 2015 |
| North Dry Rocks | NDR | 25.1297°N, 80.2938°W | 2006, 2014, 2015 |
| North North Dry Rocks | NNDR | 25. 1370°N, 80.2889°W | 2013, 2015 |
| Pickles | PI | 24.9861° N, 80.4151^o^W | 2006, 2015 |
| Sand Island | SI | 25. 0184°N, 80.3666°W | 2014, 2015 |
| Watsons | WR | 25.1875°N, 80. 2415°W | 2006, 2007, 2015 |
| White Banks Dry Rocks, north patch | WBDR1 | 25.0437°N, 80.3681°W | 2006, 2014, 2015 |
| White Banks Dry Rocks, south patch | WBDR2 | 25.0404°N, 80.3709°W | 2006, 2014, 2015 |

Suppl Table 2. Names, abbreviations, coordinates, and census dates for each site arranged in alphabetical order.
