# Supplementary material for: Reef-scale trends in Florida Acropora spp. abundance and the effects of population enhancement
Source: PeerJ. 2016 Sep 29;4:e2523. doi: 10.7717/peerj.2523 (PMC5047146; doi:10.7717/peerj.2523)
Supplement: Table S3 — Summary of congruent observed areas, colony densities, and number of outplants for each species over the longest interval of observation at each site. Change in density is represented as a proportion of the initial density. Information on numbers of outplants provided by Coral Restoration Foundation, the only organization performing large-scale population enhancement in the study area. These data were used to summarize overall trends and effect of population enhancement (see text, Table 2 and Fig. 4A). [file peerj-04-2523-s004.docx]

Suppl. Table 3. Summary of congruent observed areas, colony densities, and number of outplants for each species over the longest interval of observation at each site. Change in density is represented as a proportion of the initial density. Information on numbers of outplants provided by Coral Restoration Foundation, the only organization performing large-scale population enhancement in the study area. These data were used to summarize overall trends and effect of population enhancement (see text, Table 2, Fig 4a).

|  |  | |  | *A. cervicornis* | | | | | | *A. palmata* | | | | | |
| --- | --- | --- | --- | --- | --- | --- | --- | --- | --- | --- | --- | --- | --- | --- | --- |
| Reef | | Years | Congr Area (ha) | #Ac-Early | AcDens-Early | #Ac-Late | AcDens-Late | **Change AcDens** | # Ac Outplants | #Ap-Early | ApDens-Early | #Ap-Late | ApDens-Late | **Change ApDens** | # Ap Outplants |
| CF | | 05 & 15 | 4.0 | 8 | 2.0 | 21 | 5.3 | **1.6** | 1185 | 152 | 38.5 | 19 | 4.8 | **-0.9** | 66 |
| FR | | 07 & 14 | 8.0 | 8 | 1.0 | 41 | 5.1 | **4.1** | 682 | 185 | 23.2 | 63 | 7.9 | **-0.7** | 110 |
| ML | | 06 & 15 | 9.7 | 12 | 1.2 | 269 | 27.8 | **21.4** | 3071 | 225 | 23.3 | 82 | 8.5 | **-0.6** | 377 |
| NDR | | 06 & 15 | 6.0 | 3 | 0.5 | 91 | 15.2 | **29.3** | 680 | 37 | 6.2 | 138 | 23.1 | **2.7** | 170 |
| GR | | 06 & 15 | 10.6 | 33 | 3.1 | 351 | 33.1 | **9.6** | 903 | 430 | 3.2 | 50 | 4.7 | **0.5** | 0 |
| WBDR 2 | | 06 & 15 | 6.4 | 15 | 2.3 | 178 | 27.9 | **10.9** | 1307 | 0 | 0.0 | 0 | 0.0 | **0.0** | 0 |
| WBDR 1 | | 06 & 15 | 6.2 | 172 | 27.9 | 84 | 13.6 | **-0.5** | 0 | 6 | 1.0 | 0 | 0.0 | **-1.0** | 0 |
| LG | | 06 & 15 | 2.0 | 1 | 0.5 | 3 | 1.5 | **2.0** | 0 | 87 | 43.9 | 19 | 9.6 | **-0.8** | 0 |
| NNDR | | 06 & 15 | 5.5 | 0 | 0.0 | 3 | 0.5 | **----** | 0 | 5 | 0.9 | 4 | 0.7 | **-0.2** | 0 |
| PI | | 06 & 15 | 6.3 | 14 | 2.2 | 405 | 64.7 | **27.9** | 6958 | 21 | 3.4 | 67 | 10.7 | **2.2** | 688 |
| AD | | 06 & 15 | 3.2 | 3 | 1.0 | 0 | 0.0 | **-1.0** | 0 | 0 | 0.0 | 0 | 0.0 | **0.0** | 0 |
| BE | | 05 & 15 | 5.0 | 50 | 10.1 | 54 | 10.9 | **0.1** | 0 | 0 | 0.0 | 0 | 0.0 | **0.0** | 0 |
| CP | | 07 & 15 | 5.2 | 0 | 0.0 | 0 | 0.0 | **0.0** | 0 | 0 | 0.0 | 0 | 0.0 | **0.0** | 0 |
| WA | | 06 & 15 | 6.2 | 1 | 0.2 | 0 | 0.0 | **-1.0** | 0 | 143 | 23.0 | 105 | 16.9 | **-0.3** | 0 |
