# Supplementary material for: Reef-scale trends in Florida Acropora spp. abundance and the effects of population enhancement
Source: PeerJ. 2016 Sep 29;4:e2523. doi: 10.7717/peerj.2523 (PMC5047146; doi:10.7717/peerj.2523)
Supplement: Figure S1 — Images showing the outcomes (tracks and waypoints) for each census at each site. Yellow symbols represent A. palmata colonies, purple symbols represent A. cervicornis colonies, and red symbols show the outline of A. palmata ‘thickets’ (see text for explanation) with different shaped symbols used for different years. Sites are are arranged in alphabetical order. Coordinates are given in Table S2. Base imagery of the reef from GoogleEarth. [file peerj-04-2523-s005.pdf]

Supplemental Figure 1: Images showing the outcomes (tracks and waypoints) for each census at each site. Yellow symbols represent A.palmata colonies, purple symbols represent A.cervicornis colonies, and red symbols show the outline of A.palmata ‘thickets’ (see text for explanation) with different shaped symbols used for different years. Sites are are arranged in alphabetical order. Coordinates are given in Suppl. Table 2. Base imagery of the reef from GoogleEarth.

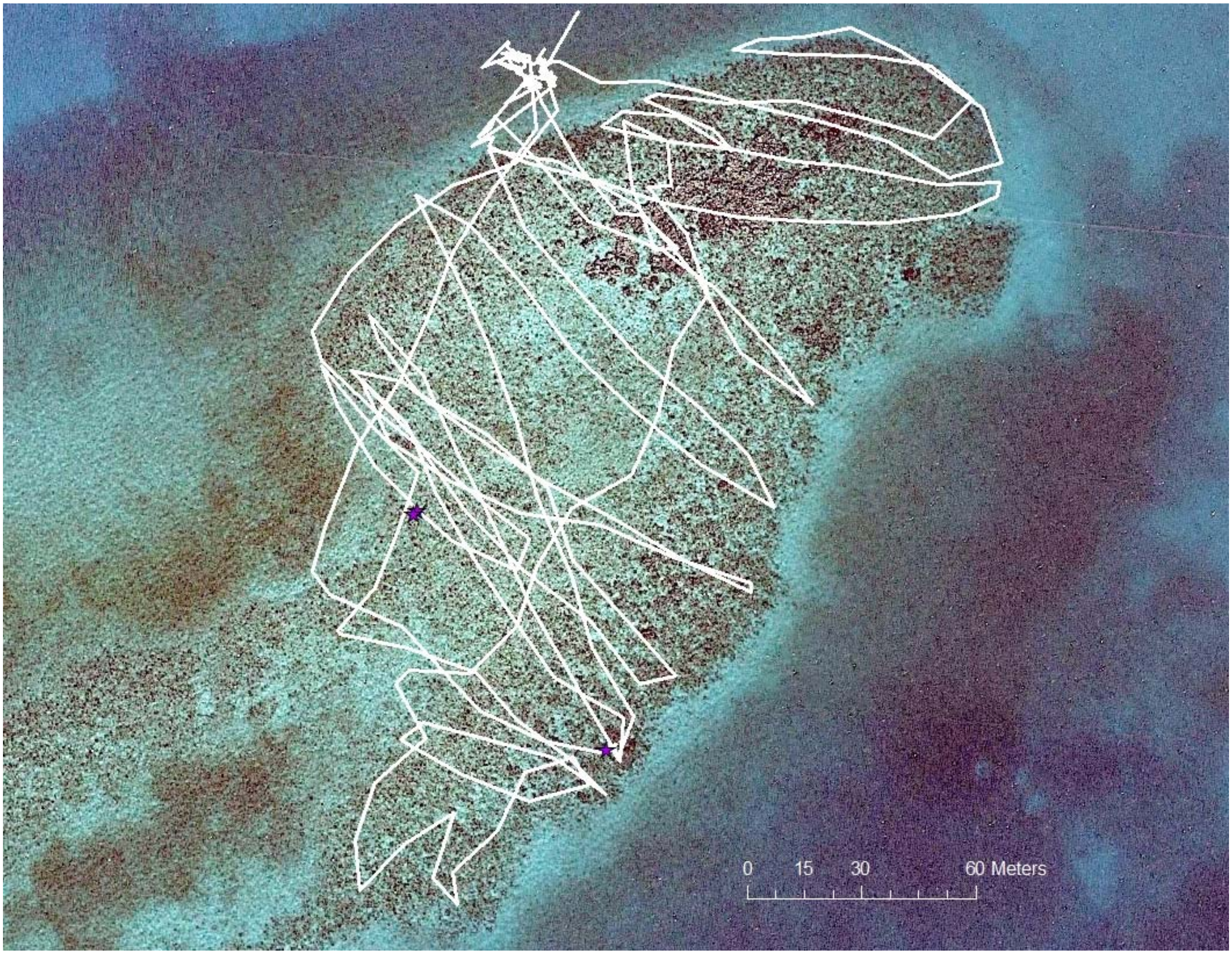


Admiral Reef 2005

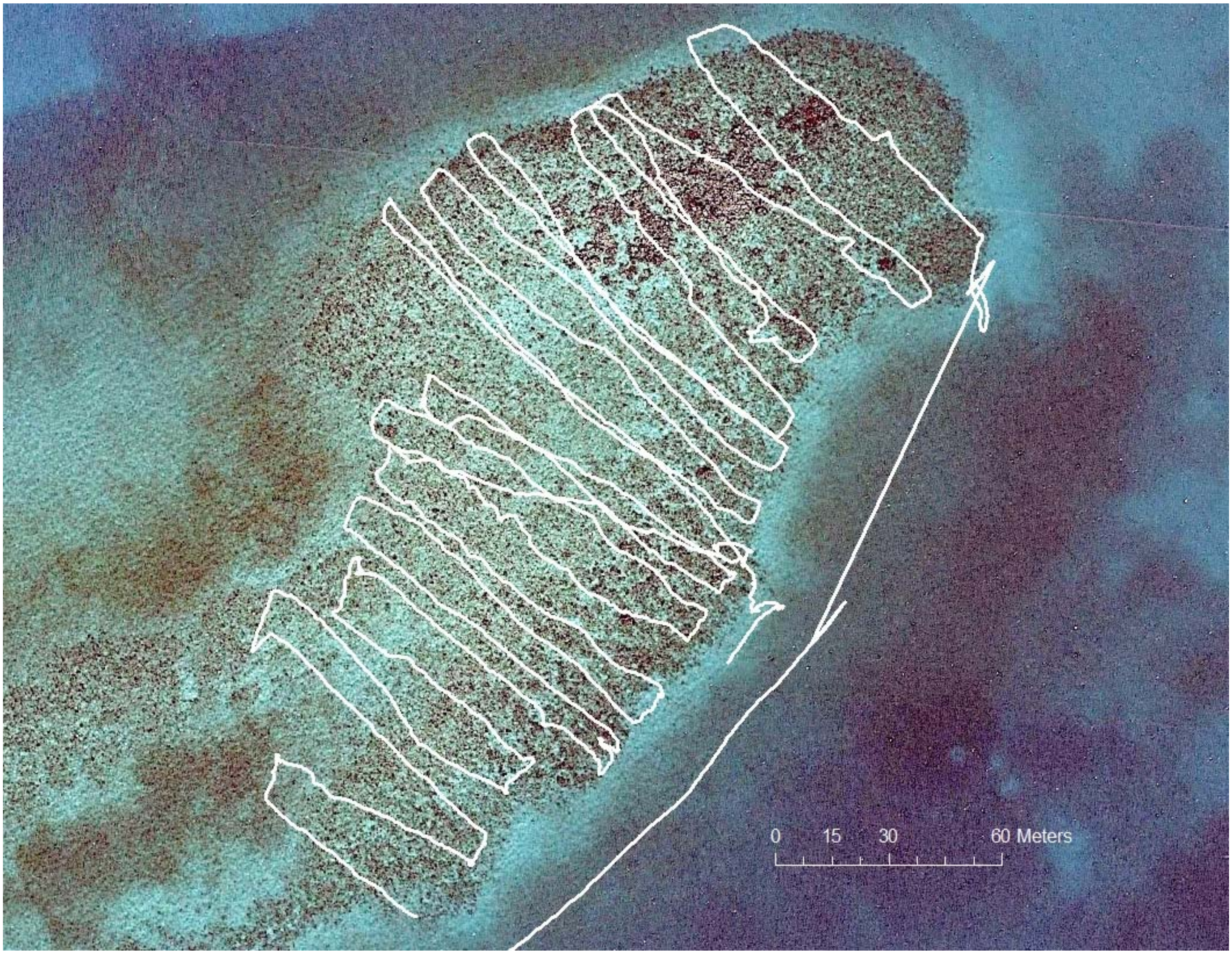


Admiral Reef 2015

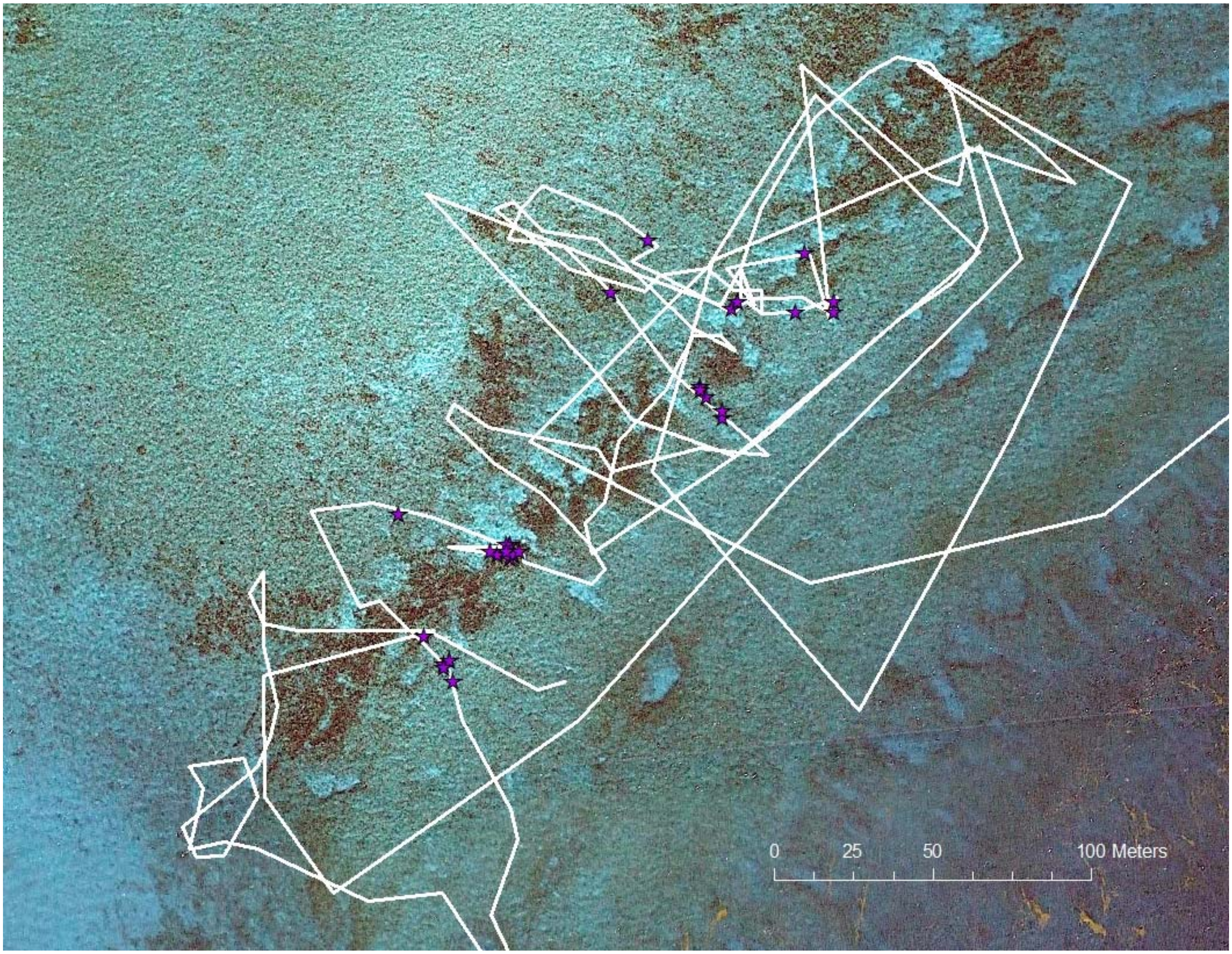


Belgium Reef 2005

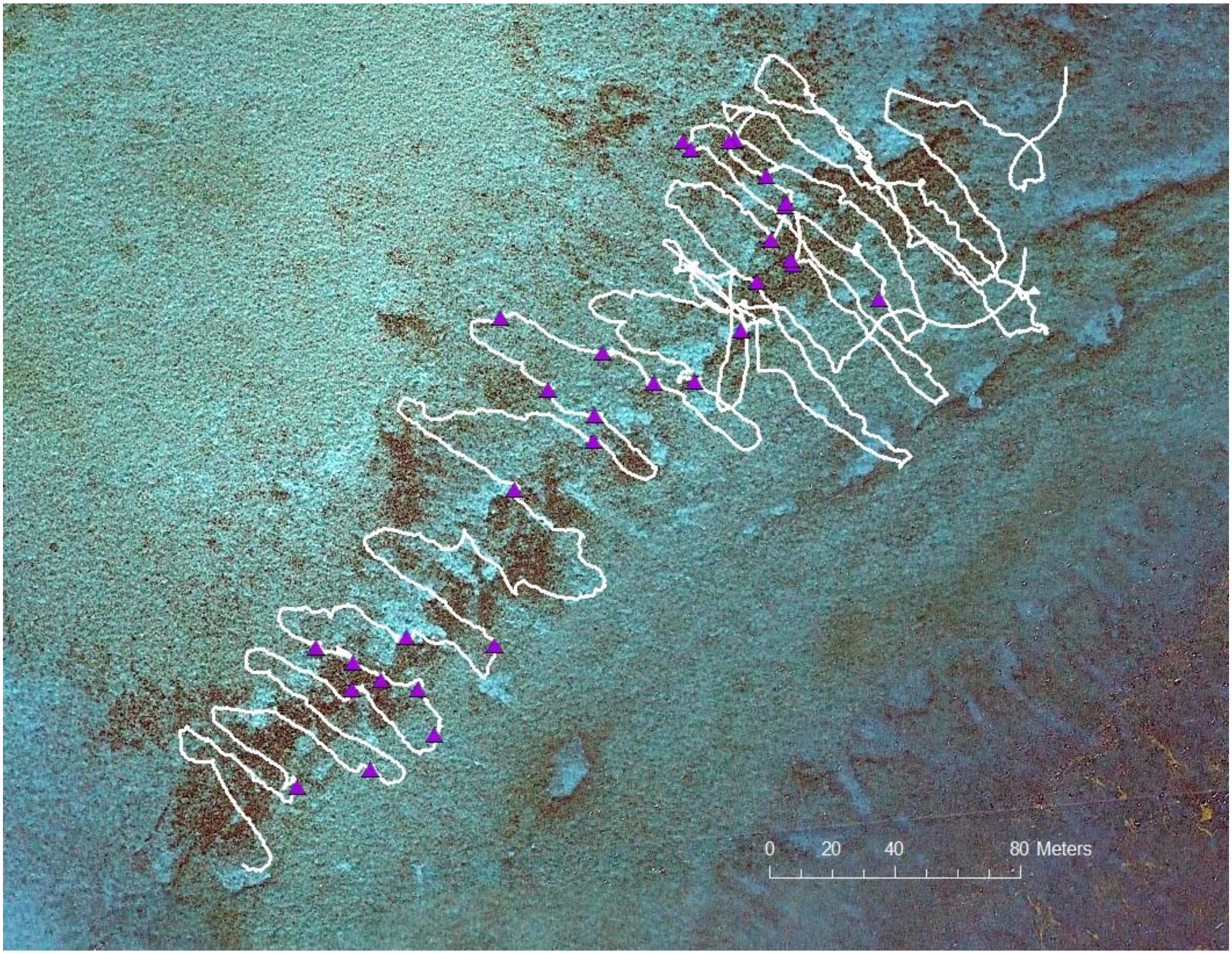


Belgium Reef 2015

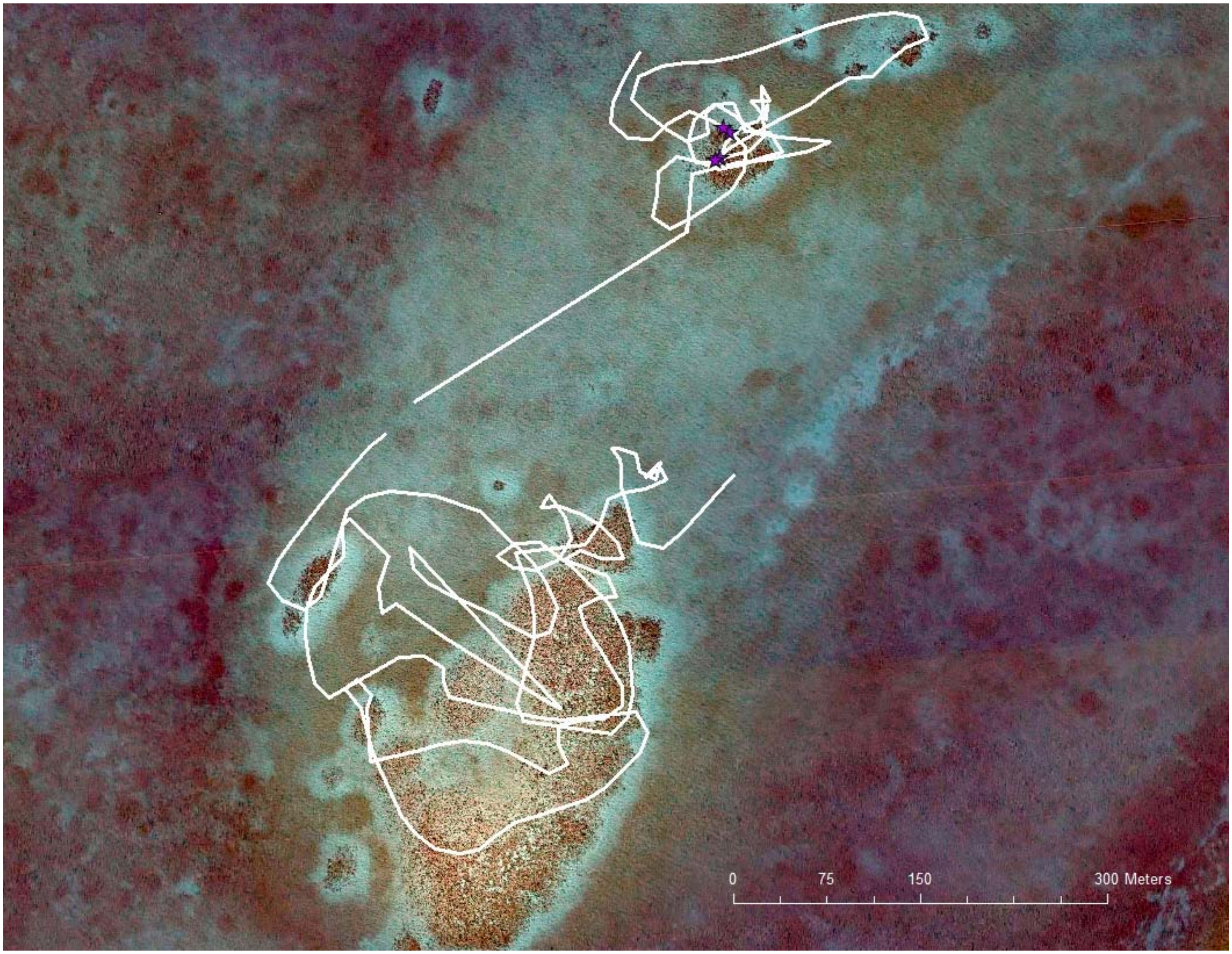


Cannon Patch 2007

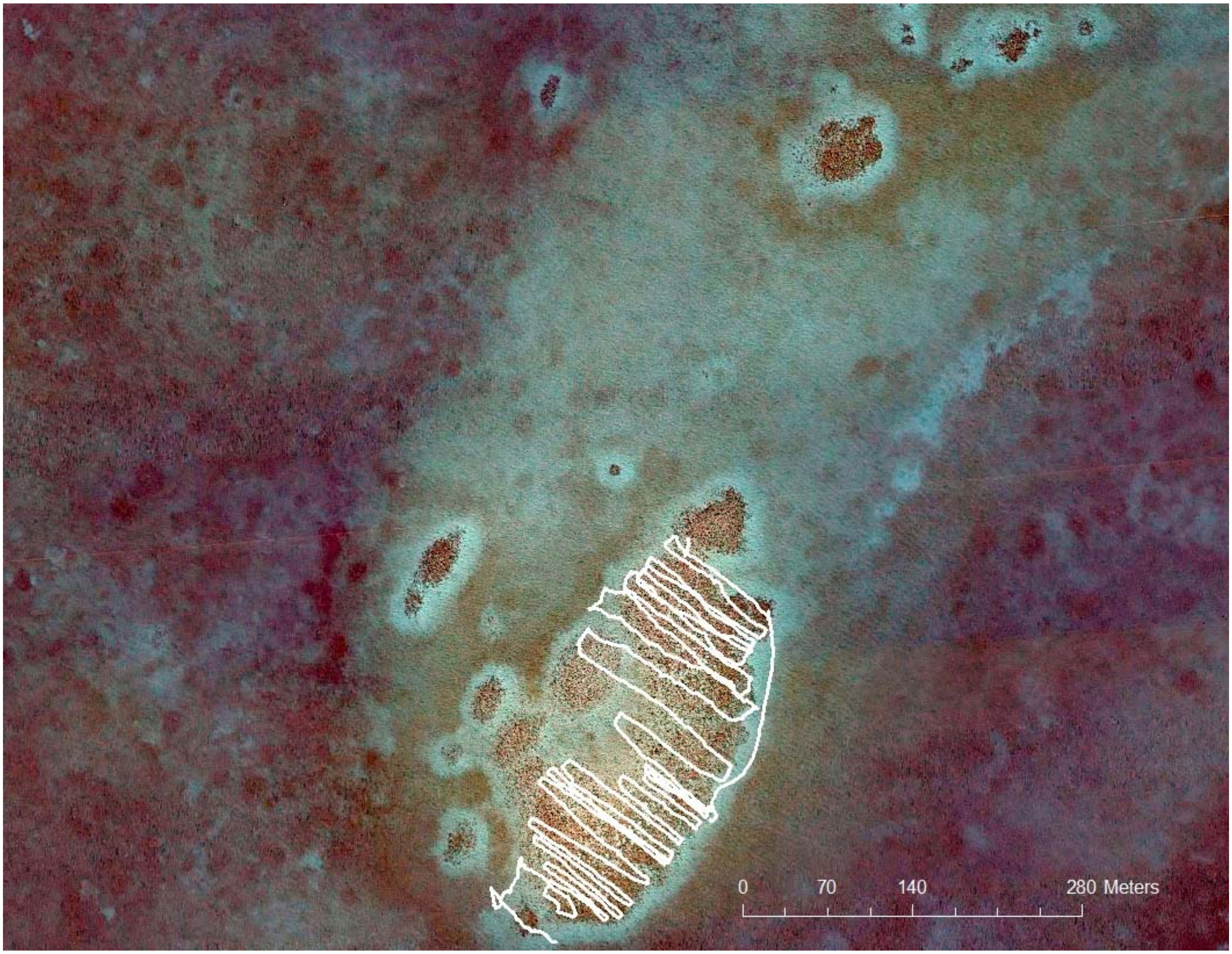


Cannon Patch 2015

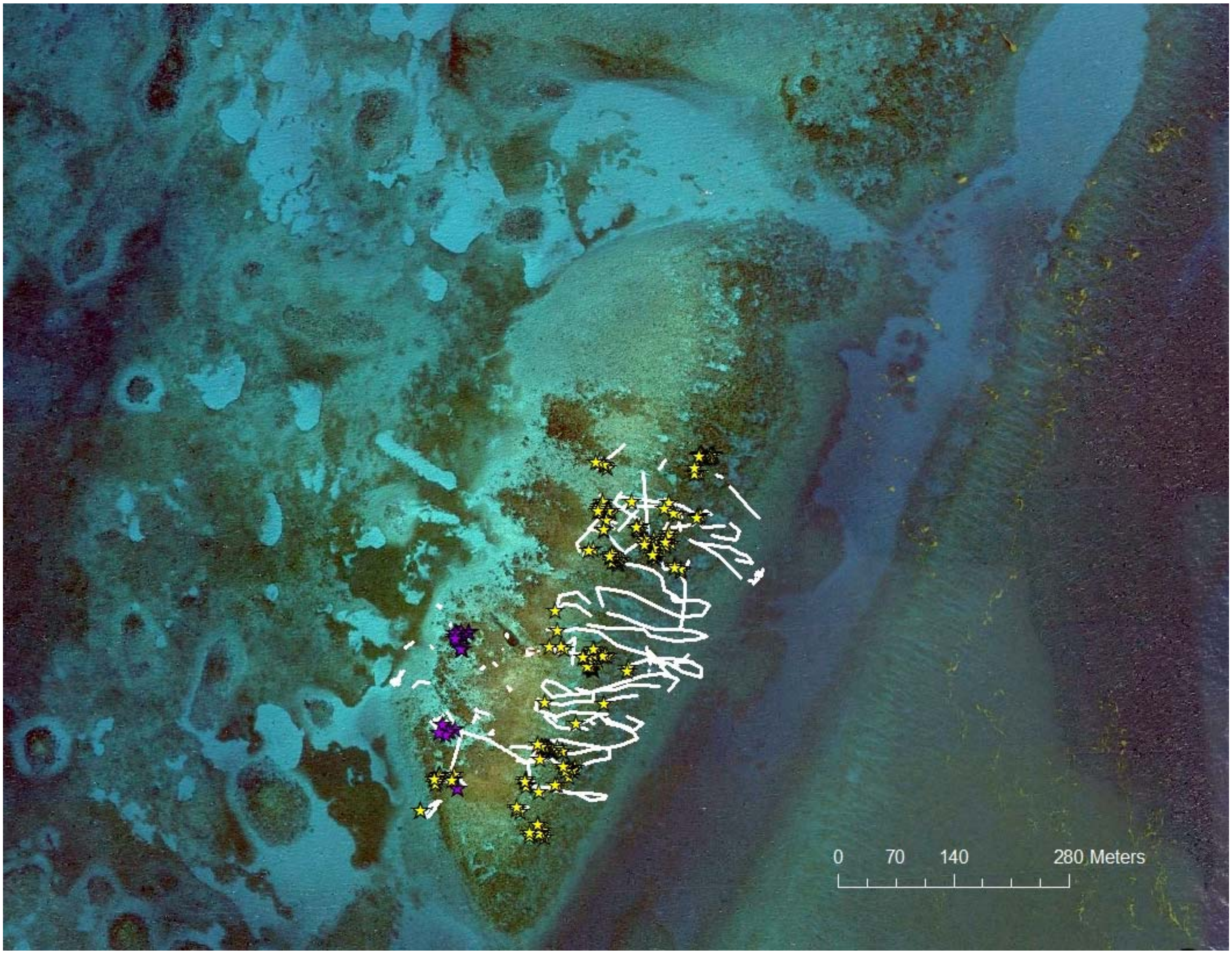


Carysfort Reef 2005

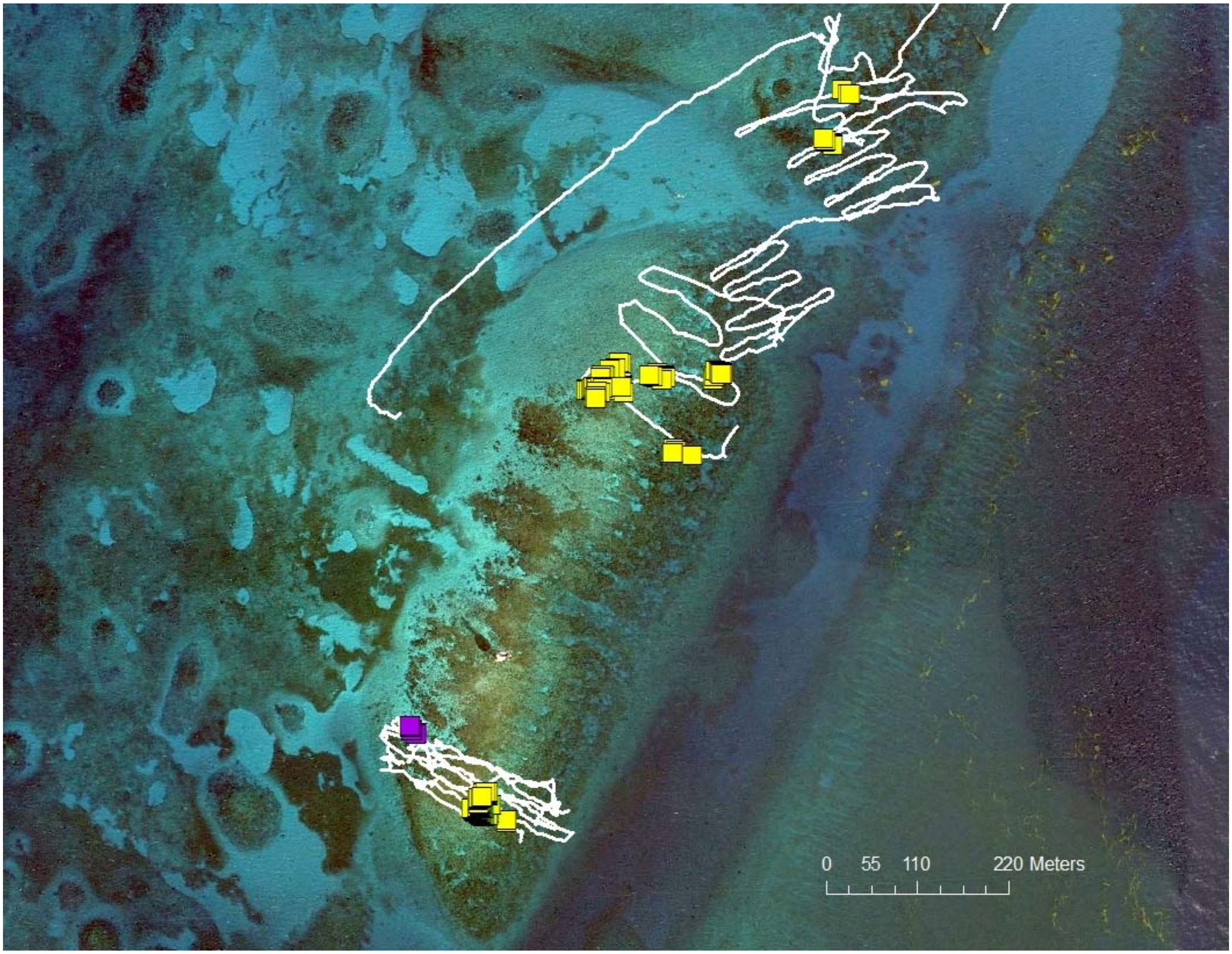


Carysfort Reef 2014

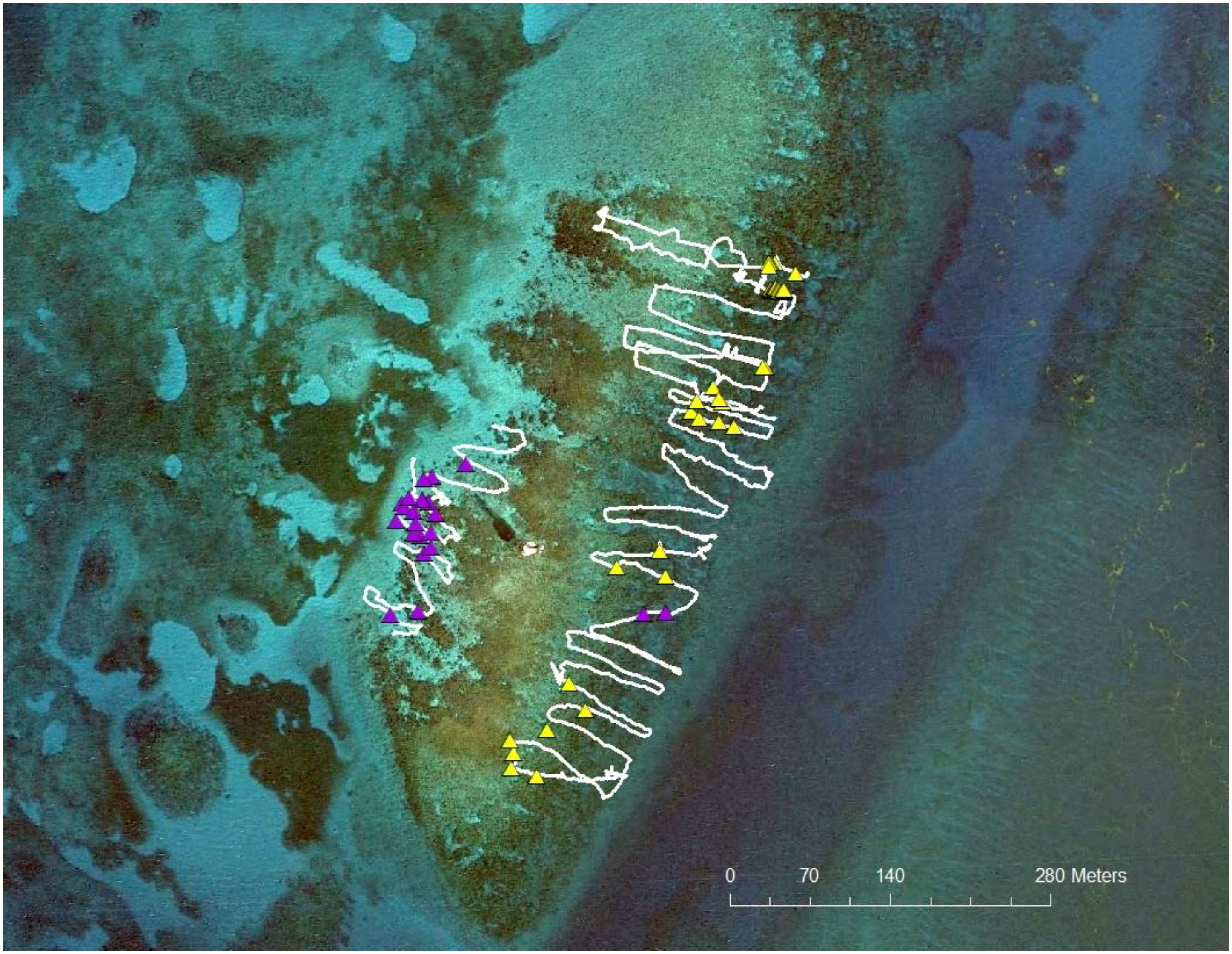


Carysfort Reef 2015

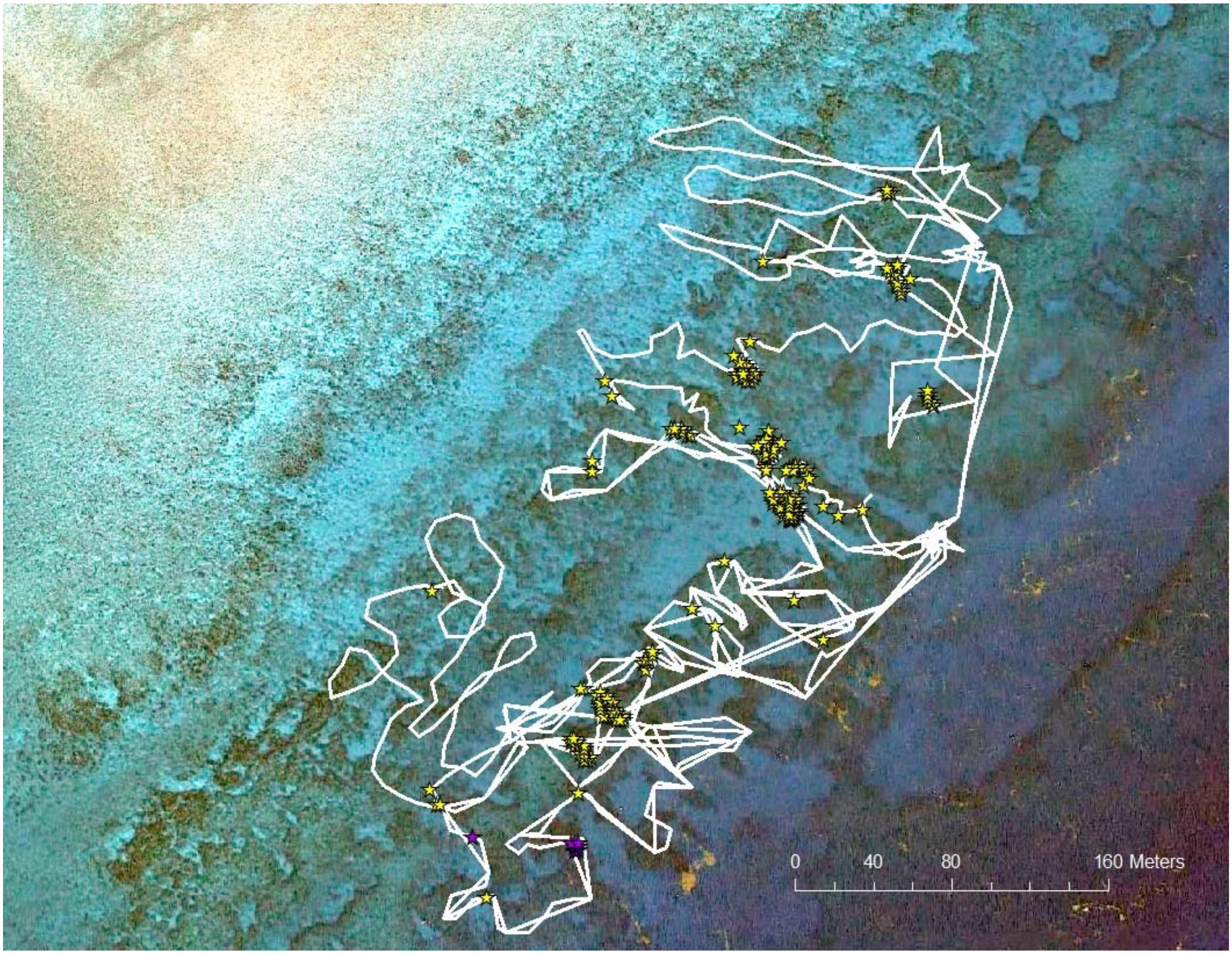


French Reef 2007

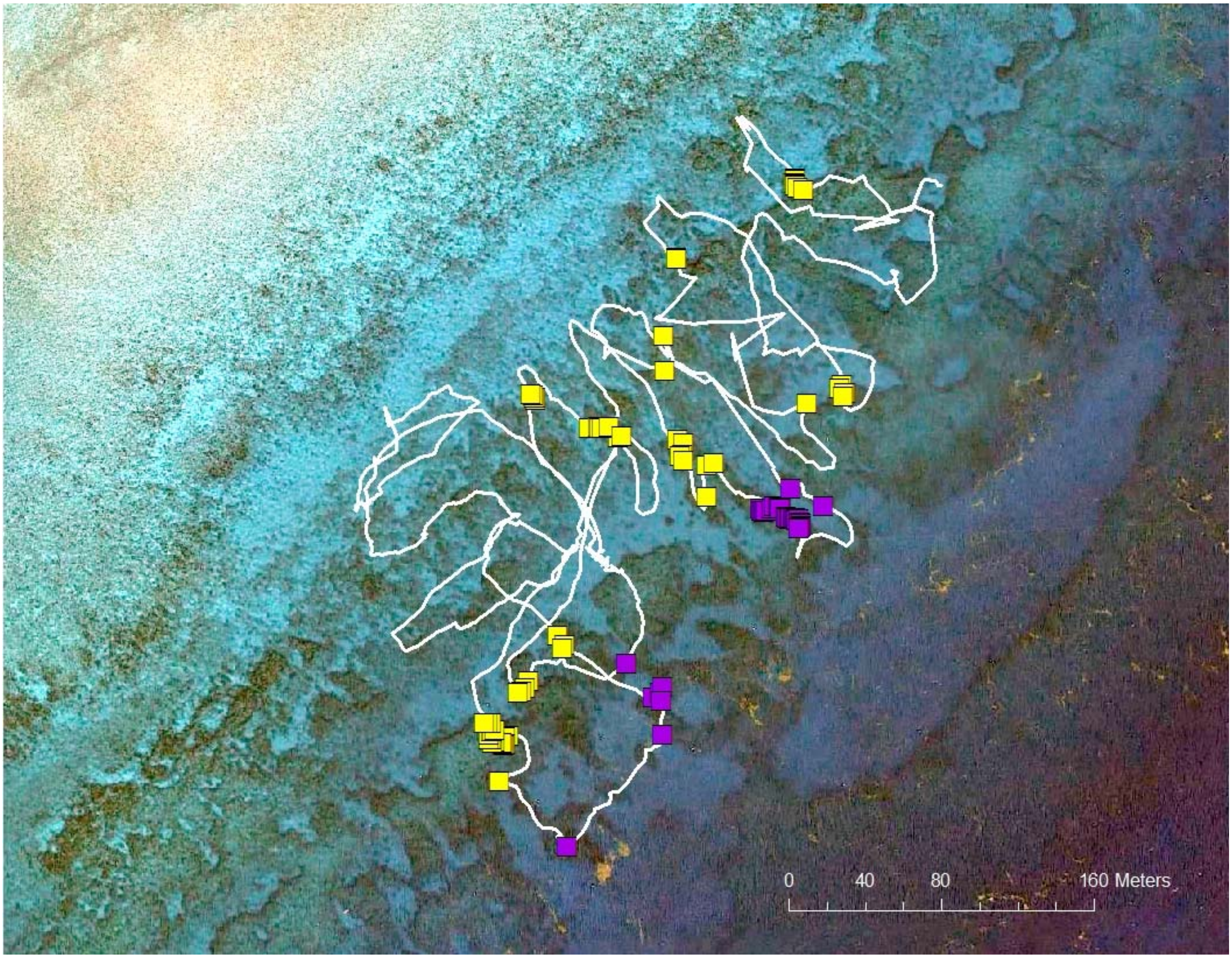


French Reef 2014

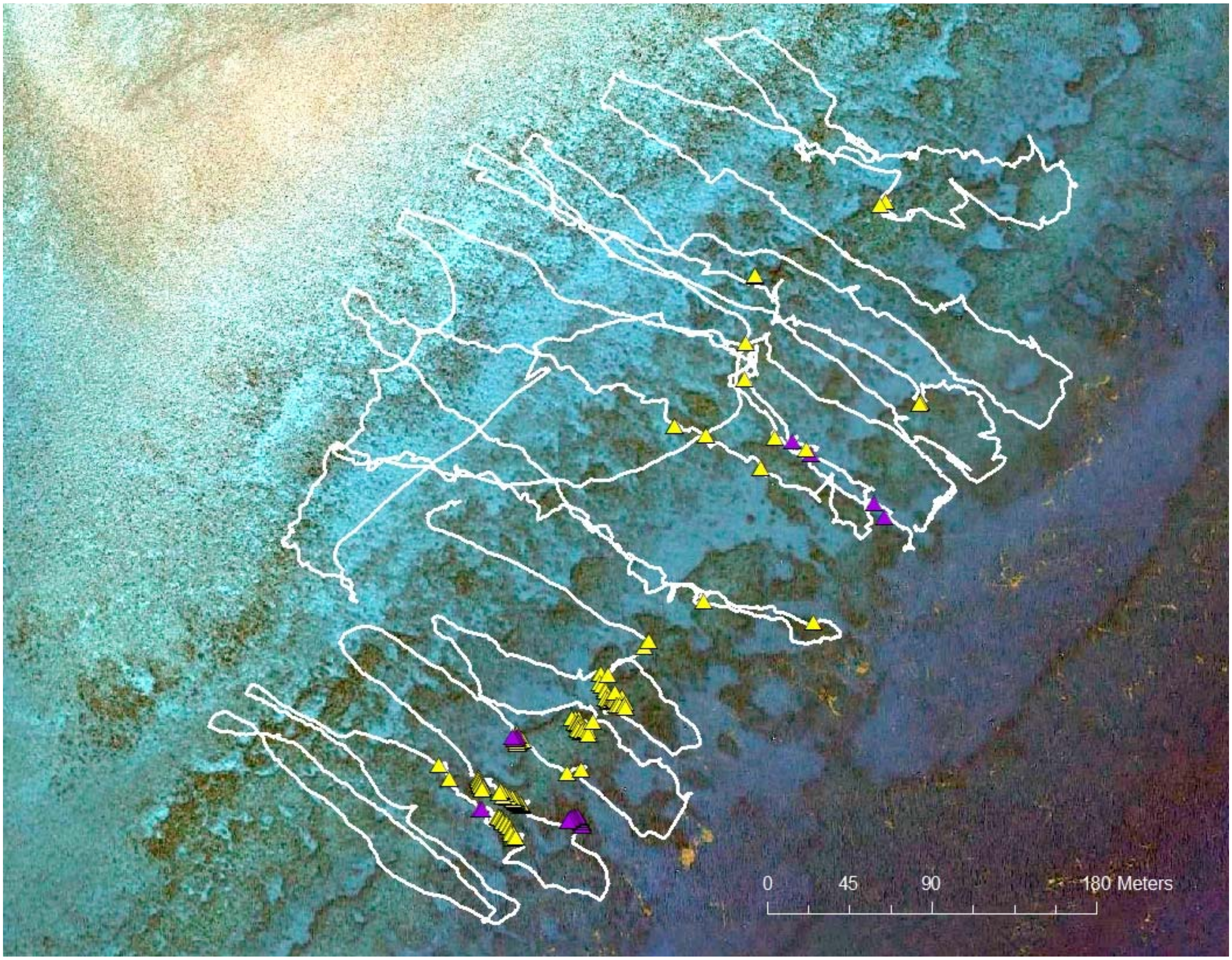


French Reef 2015

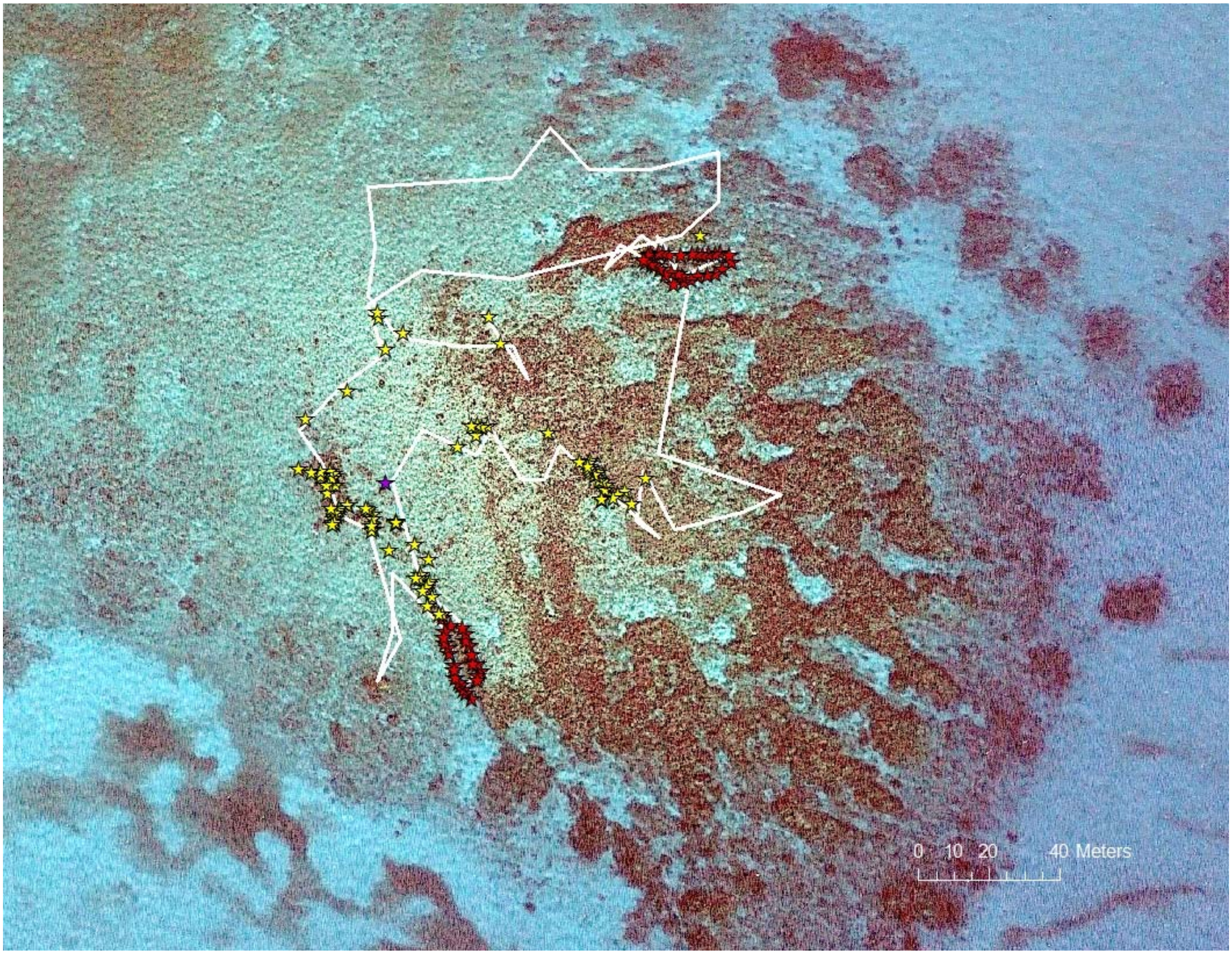


Little Grecian 2006

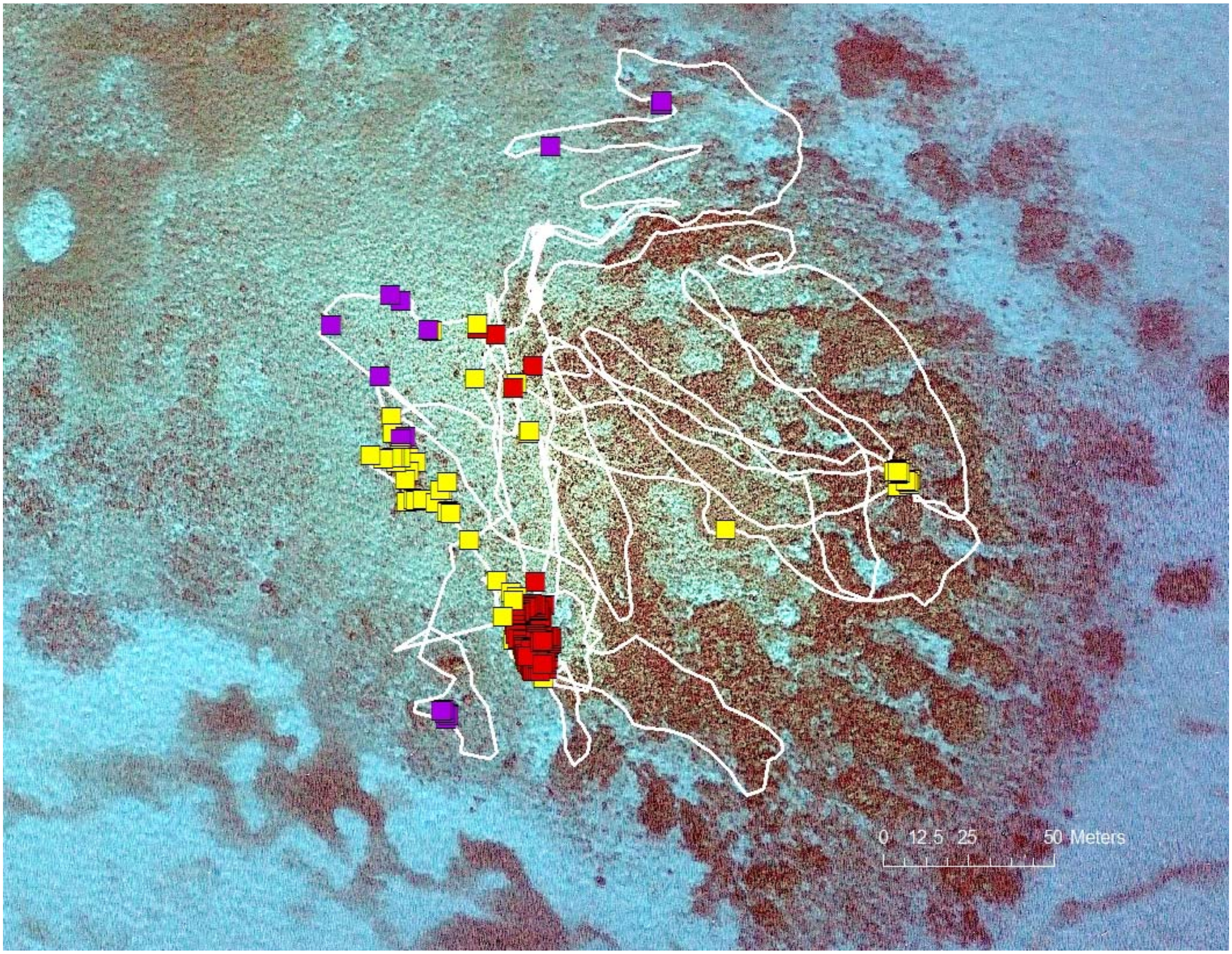


Little Grecian 2013

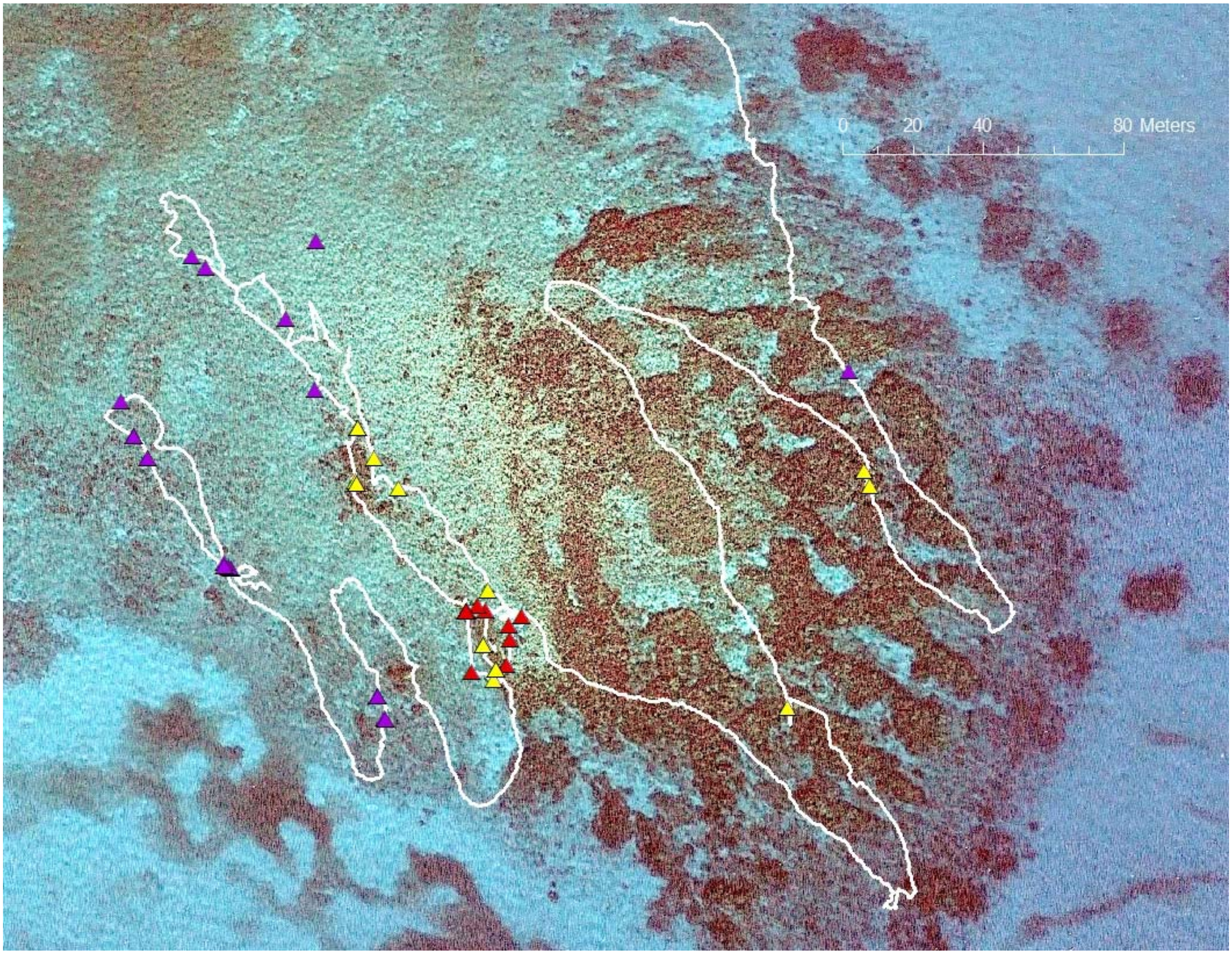


Little Grecian 2015

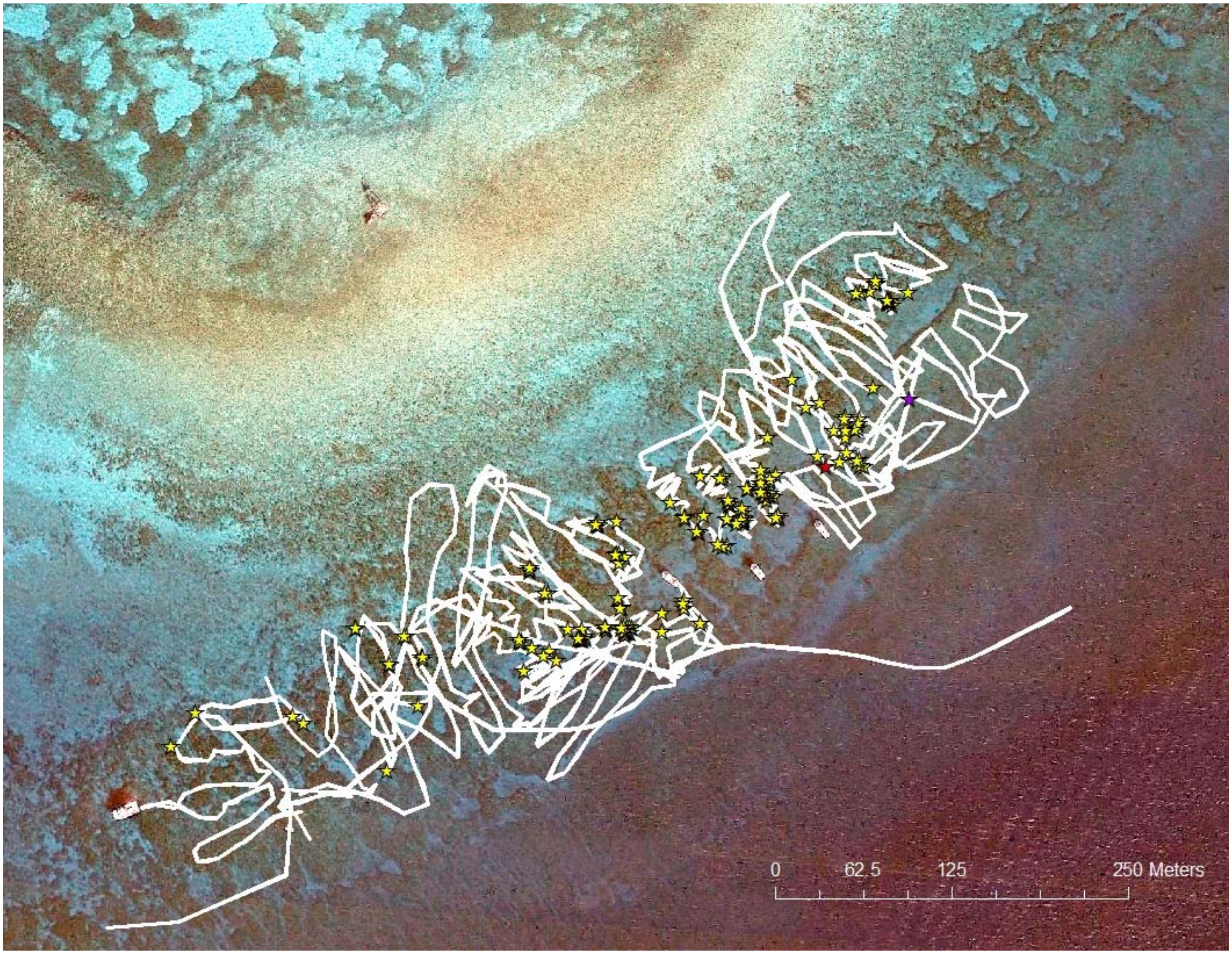


Molasses Reef 2006

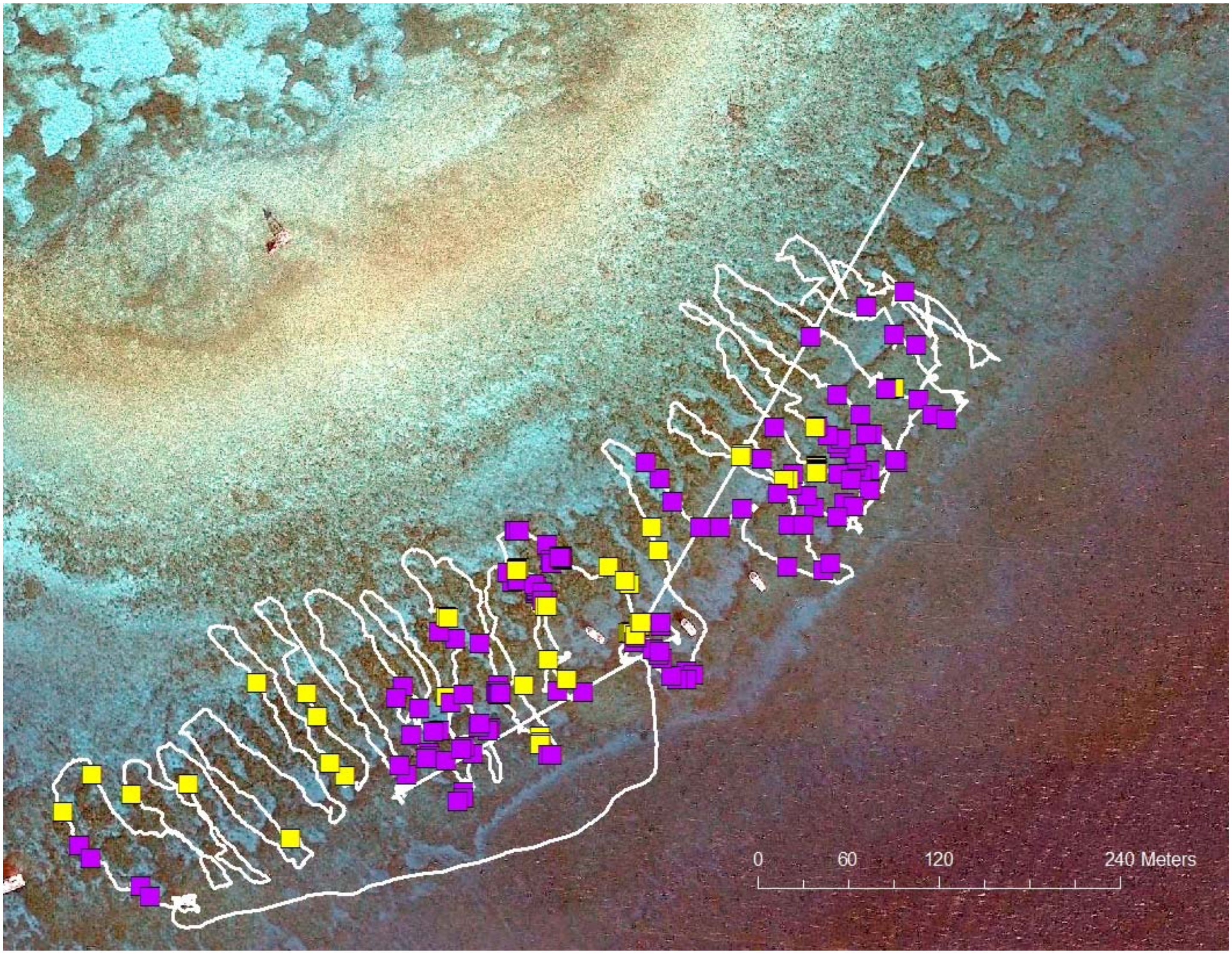


Molasses Reef 2014

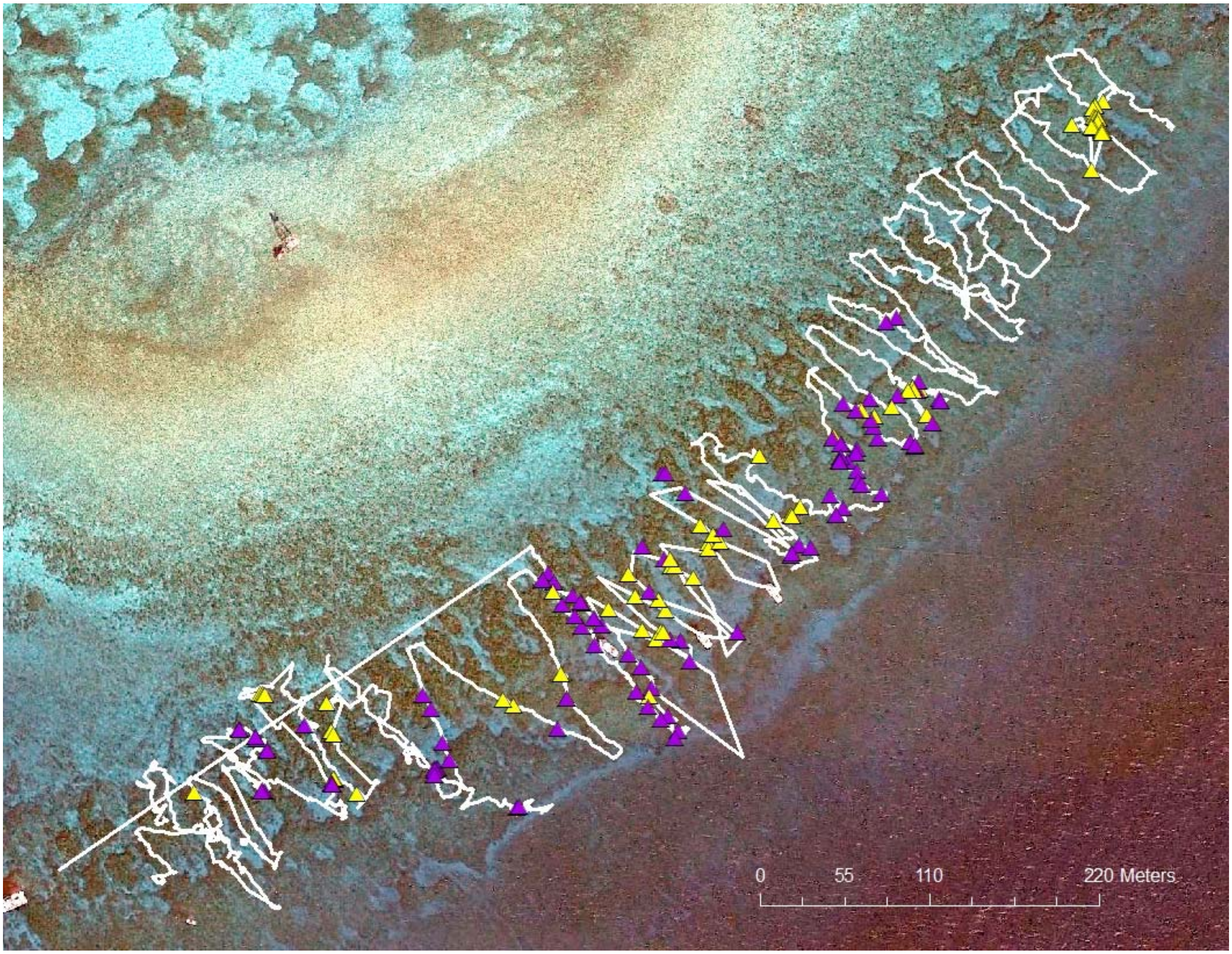


Molasses Reef 2015

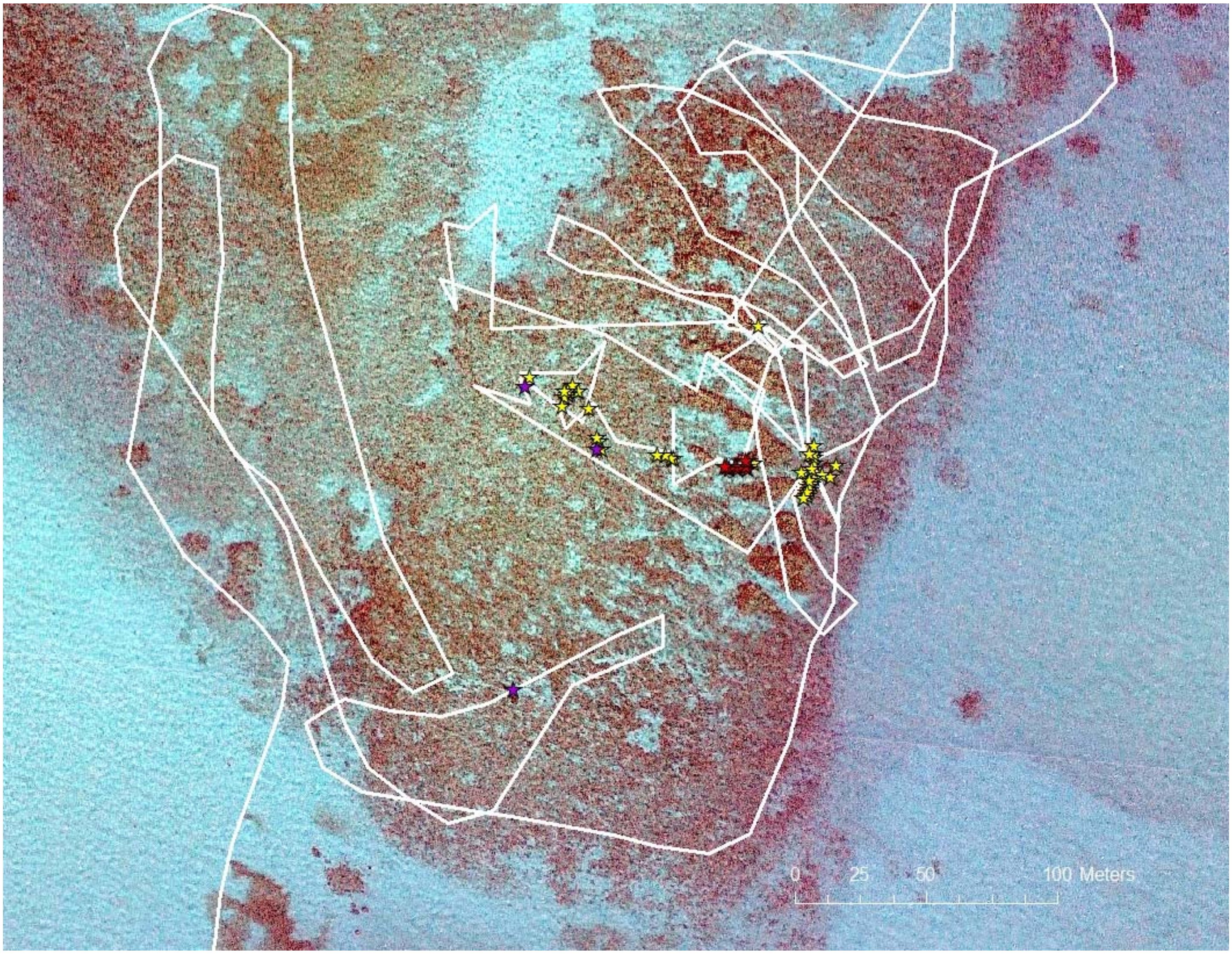


North Dry Rocks 2006

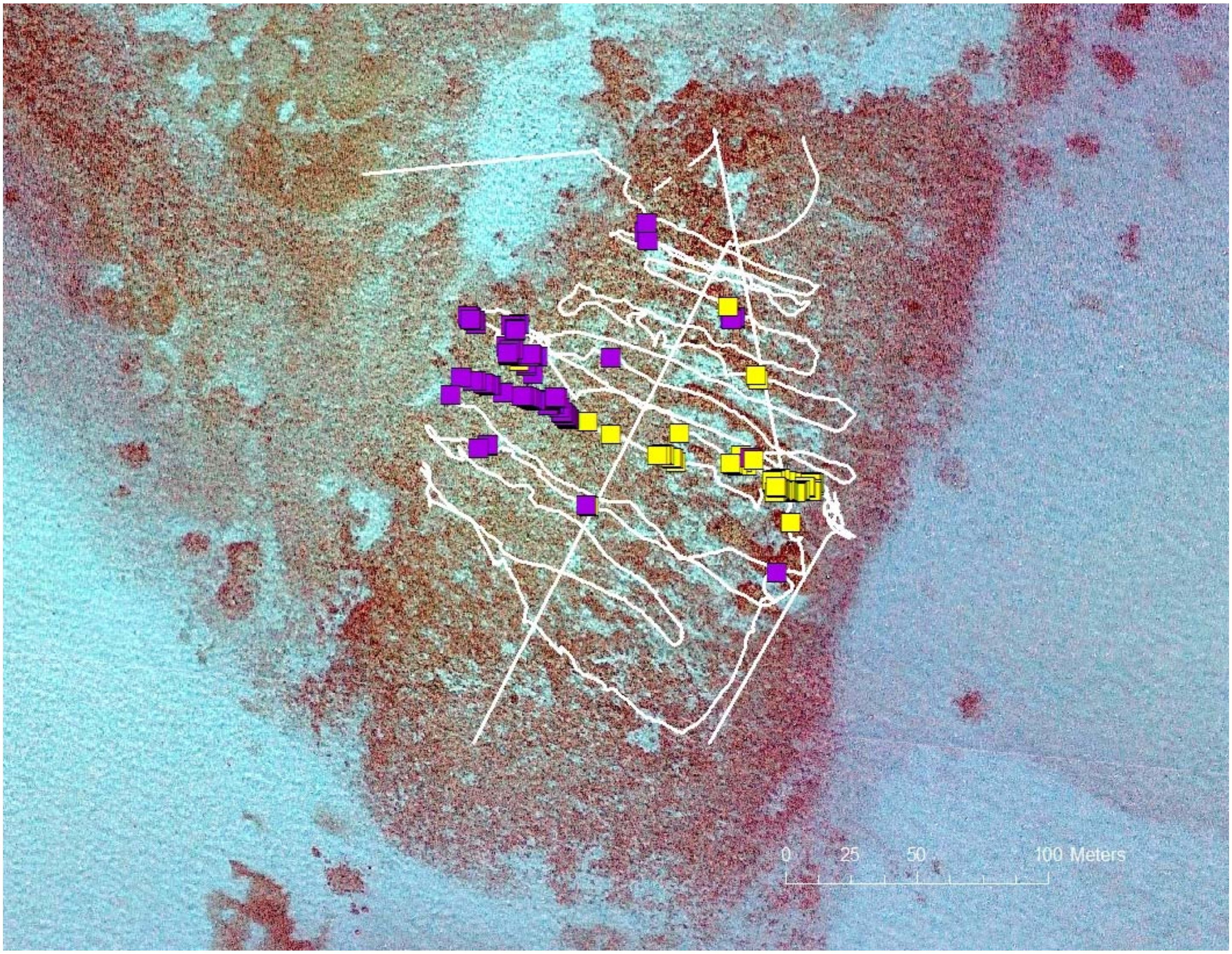


North Dry Rocks 2014

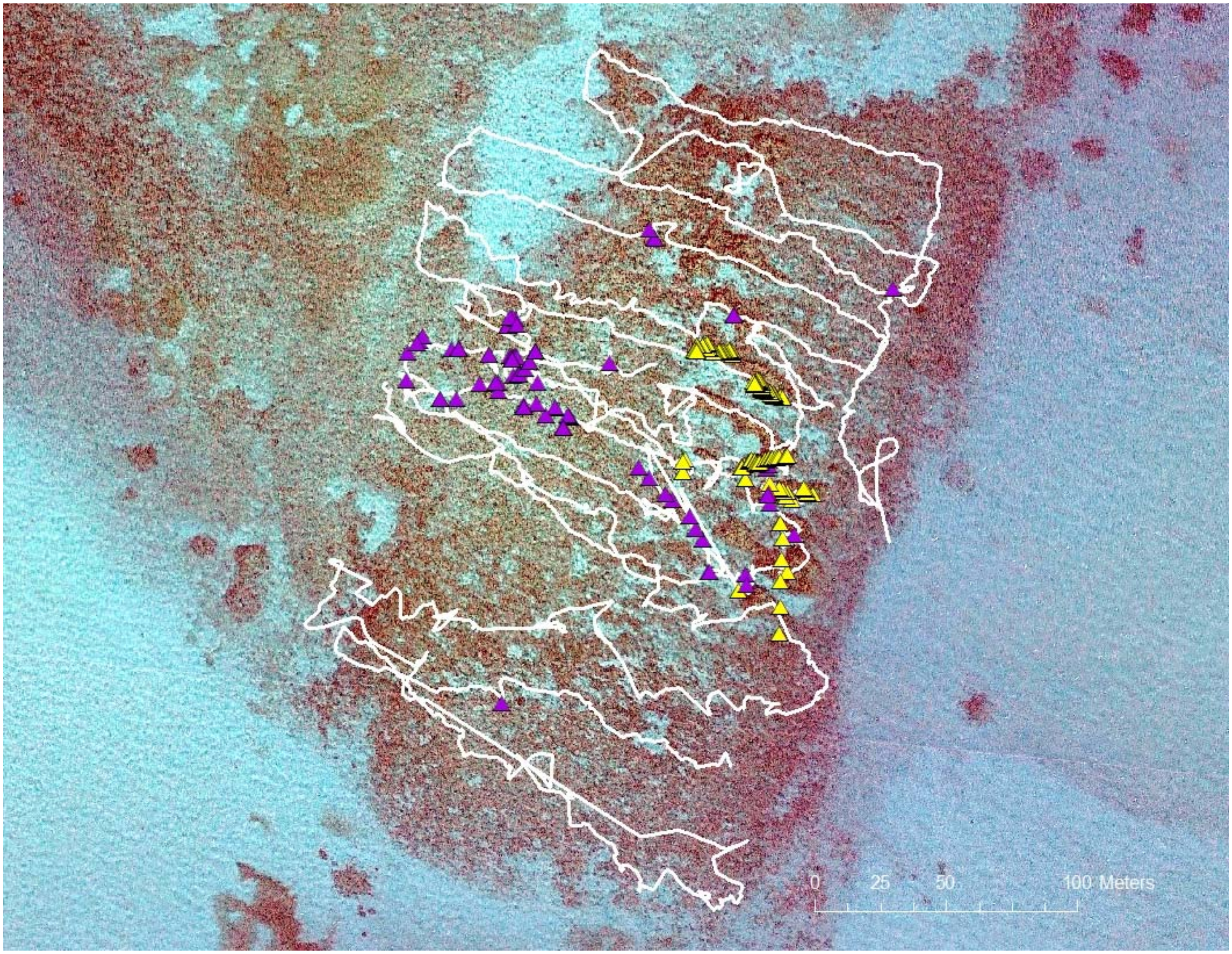


North Dry Rocks 2015

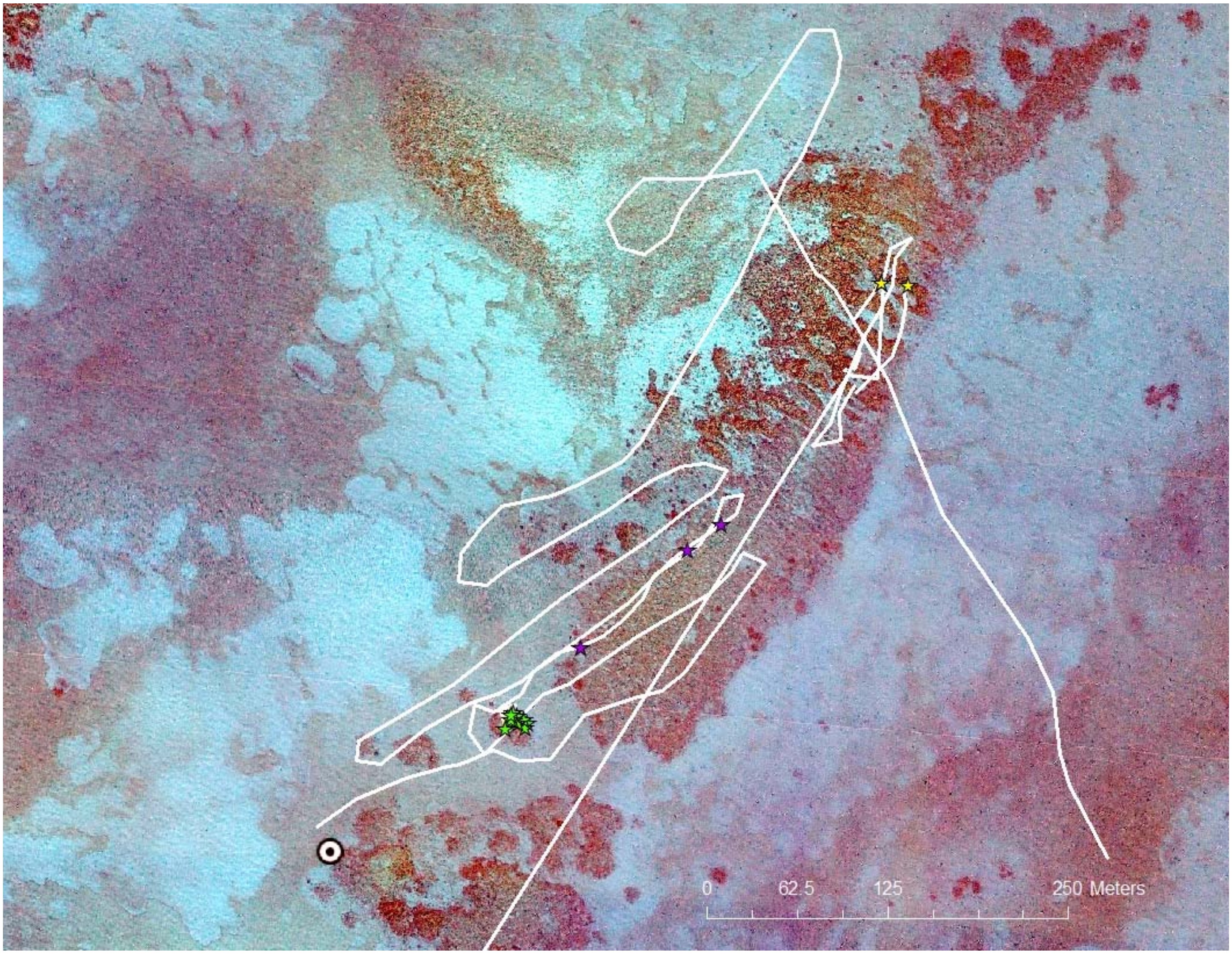


North North Dry Rocks 2006

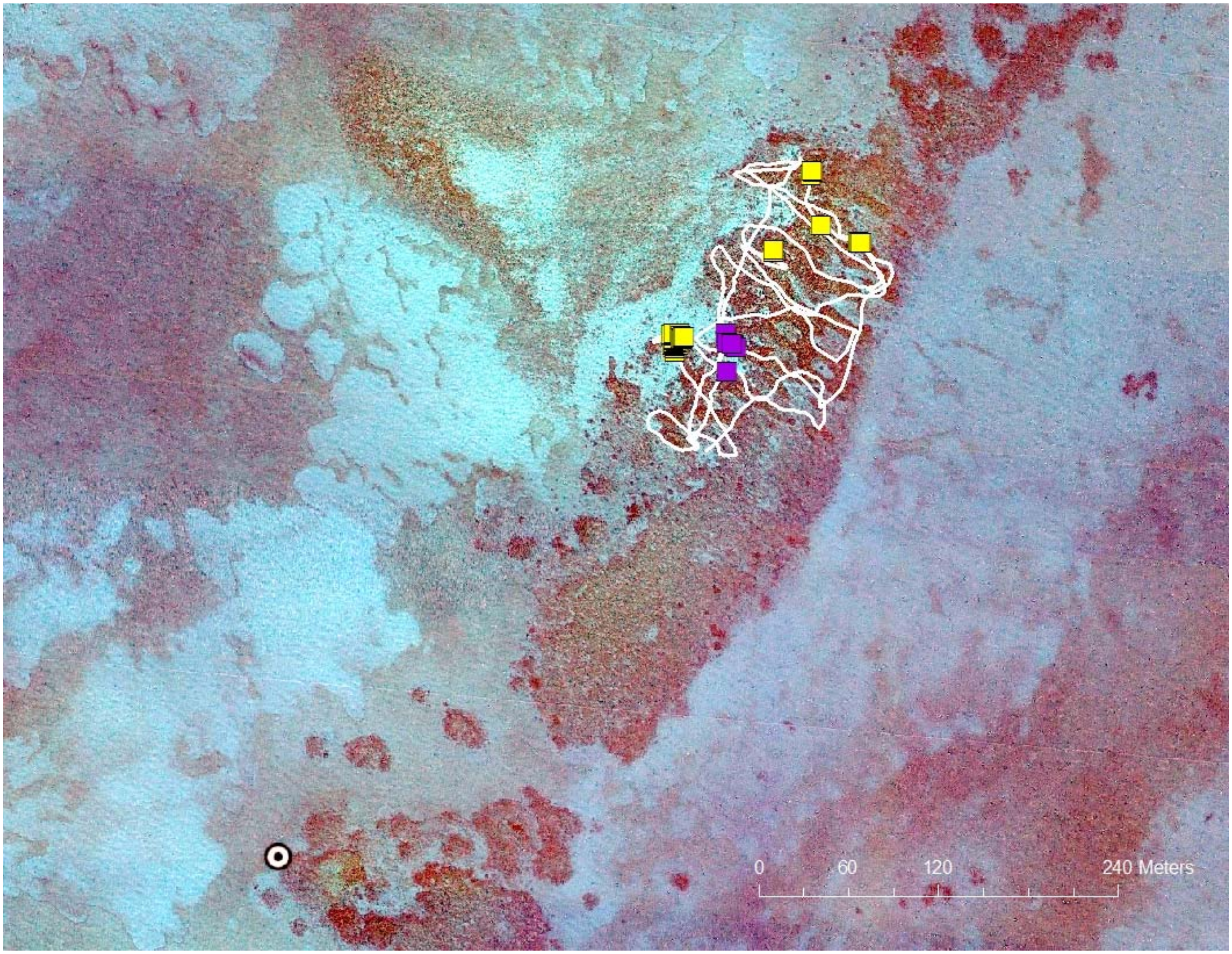


North North Dry Rocks 2013

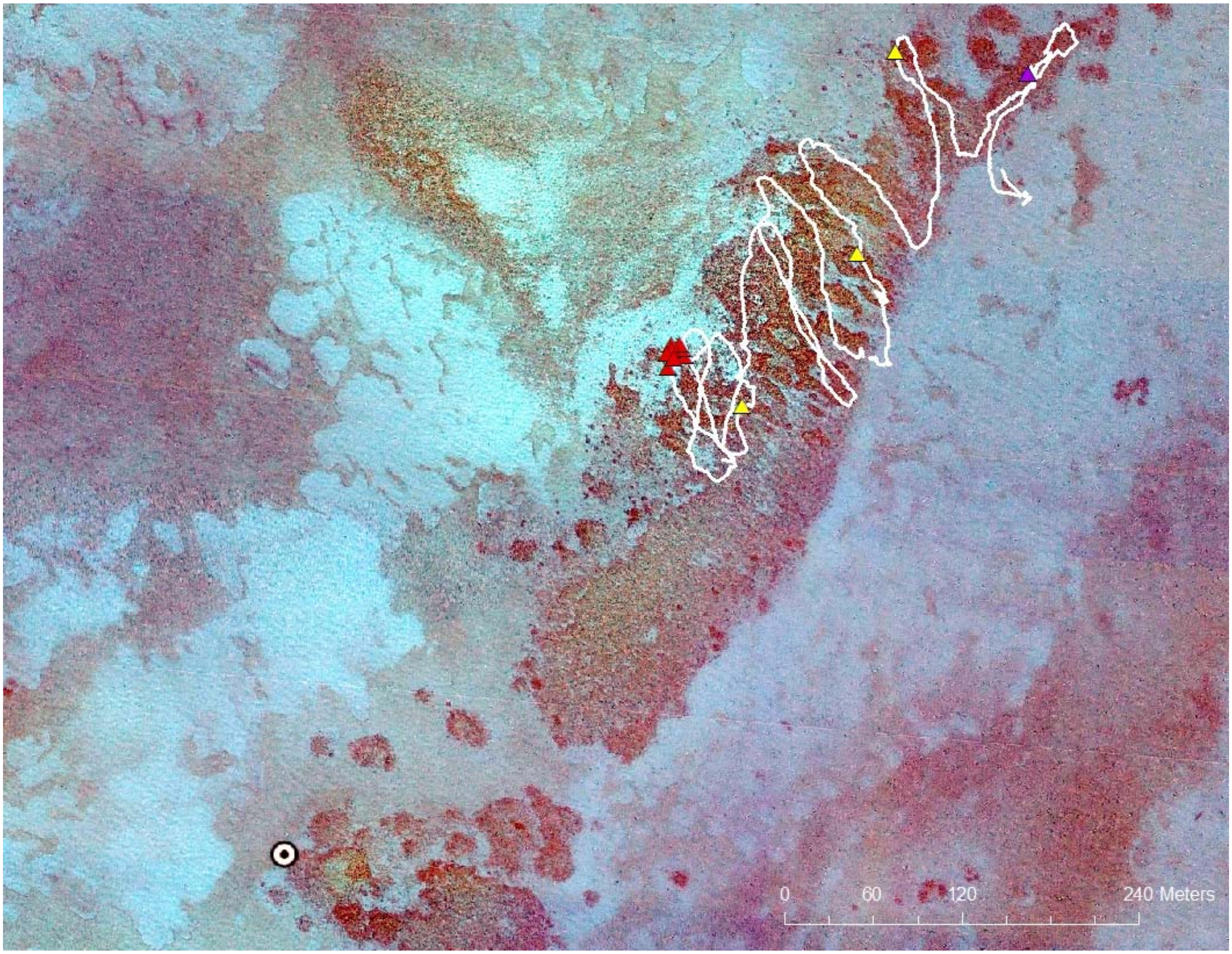


North North Dry Rocks 2015

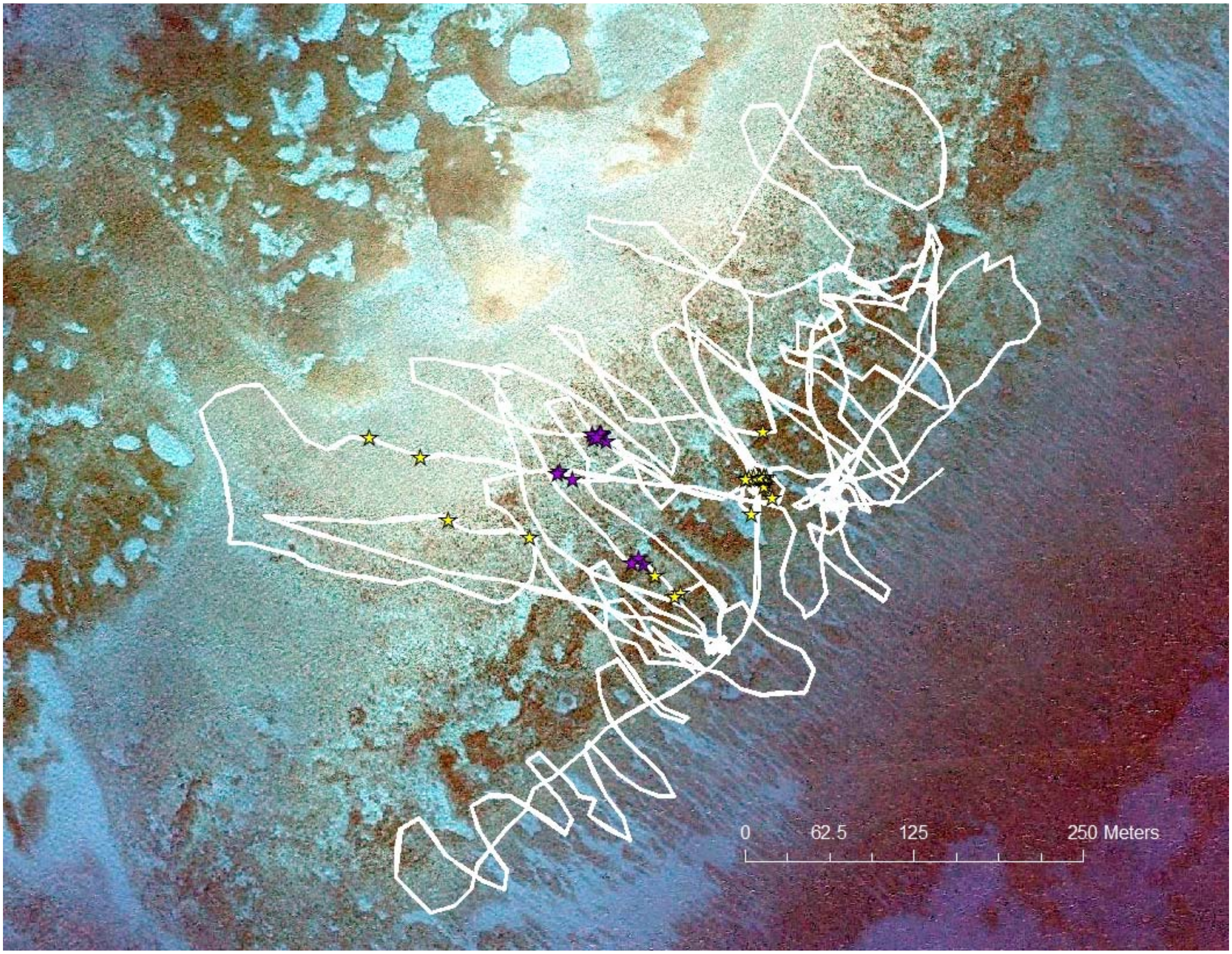


Pickles Reef 2006

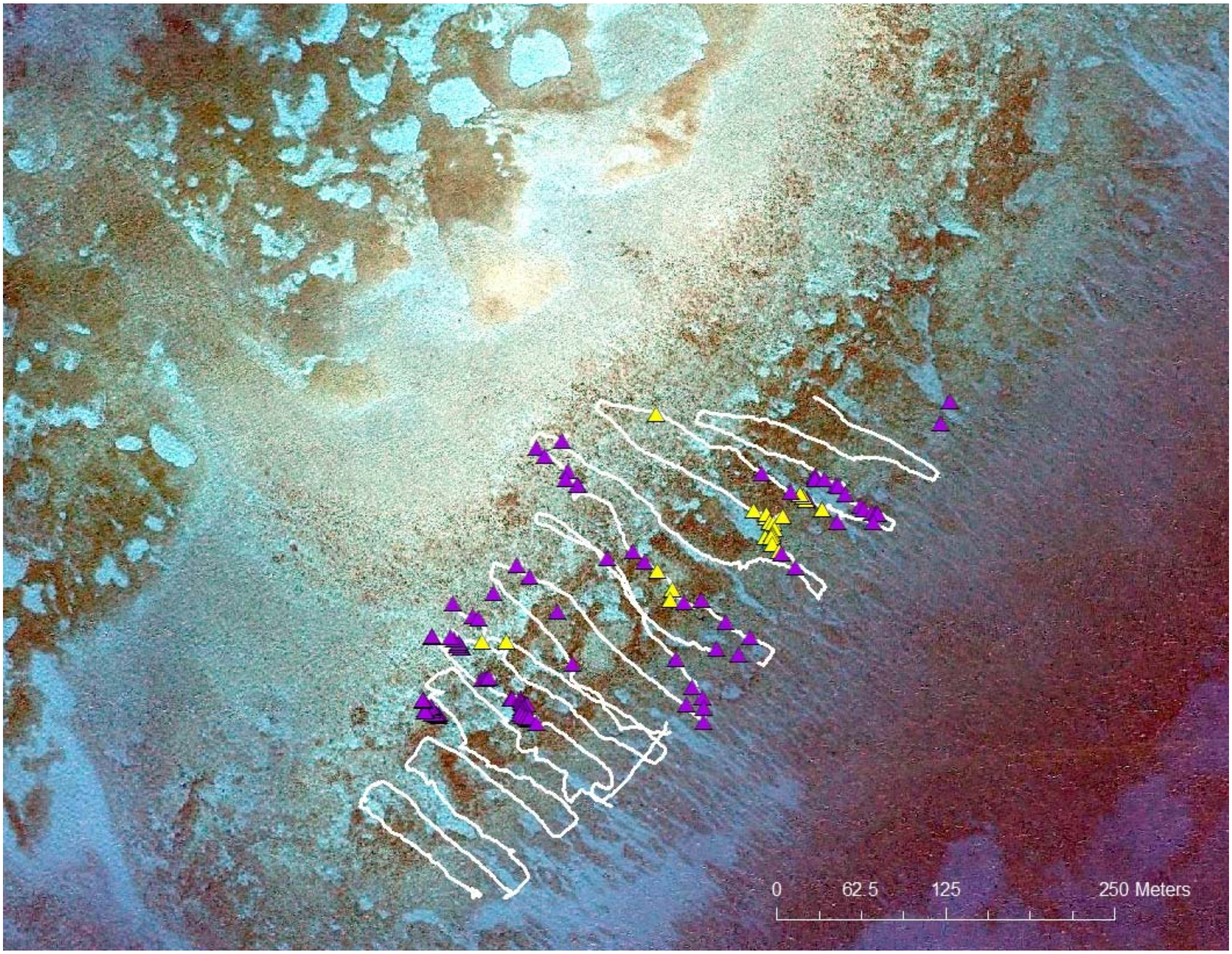


Pickles Reef 2015

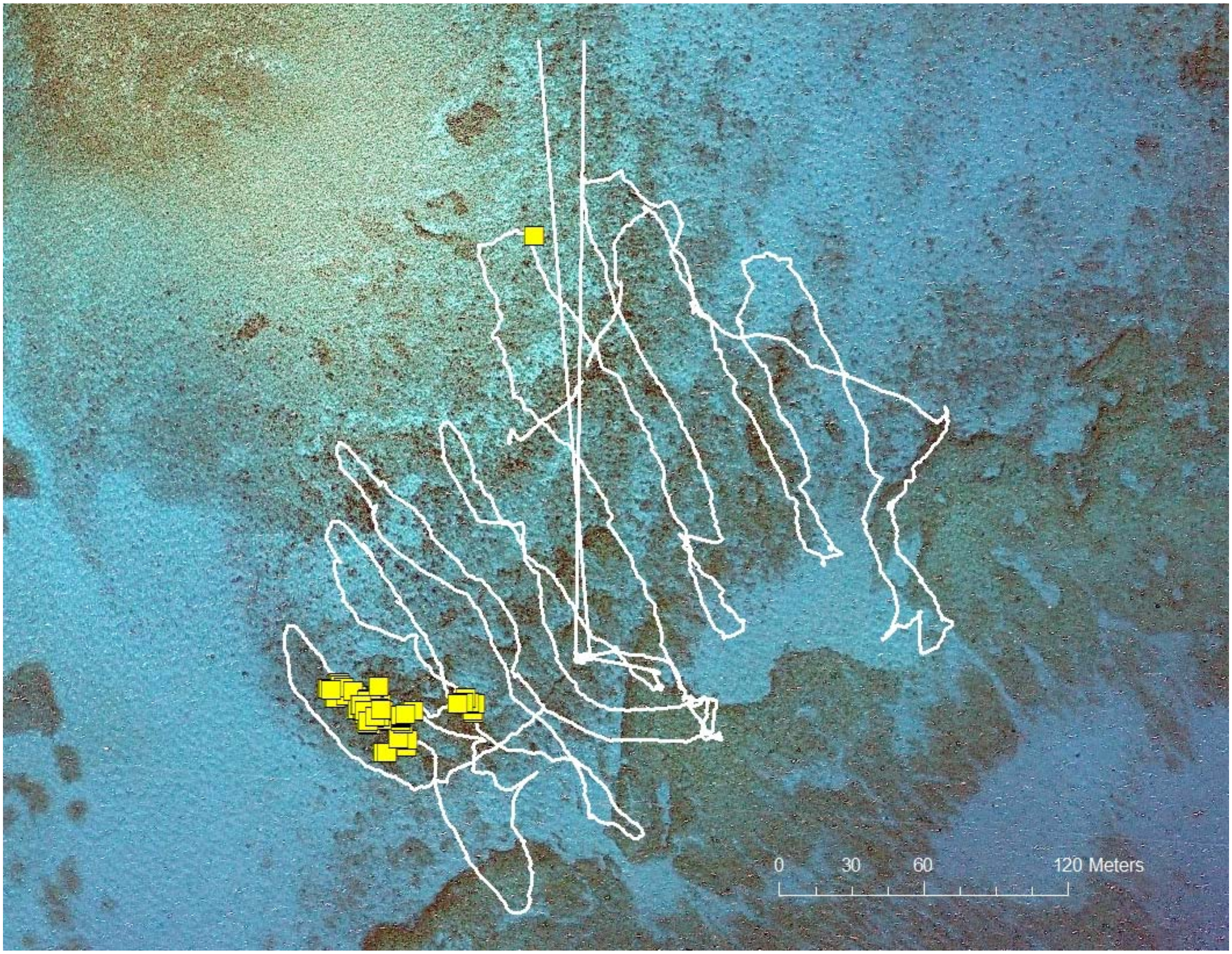


Sand Island 2014

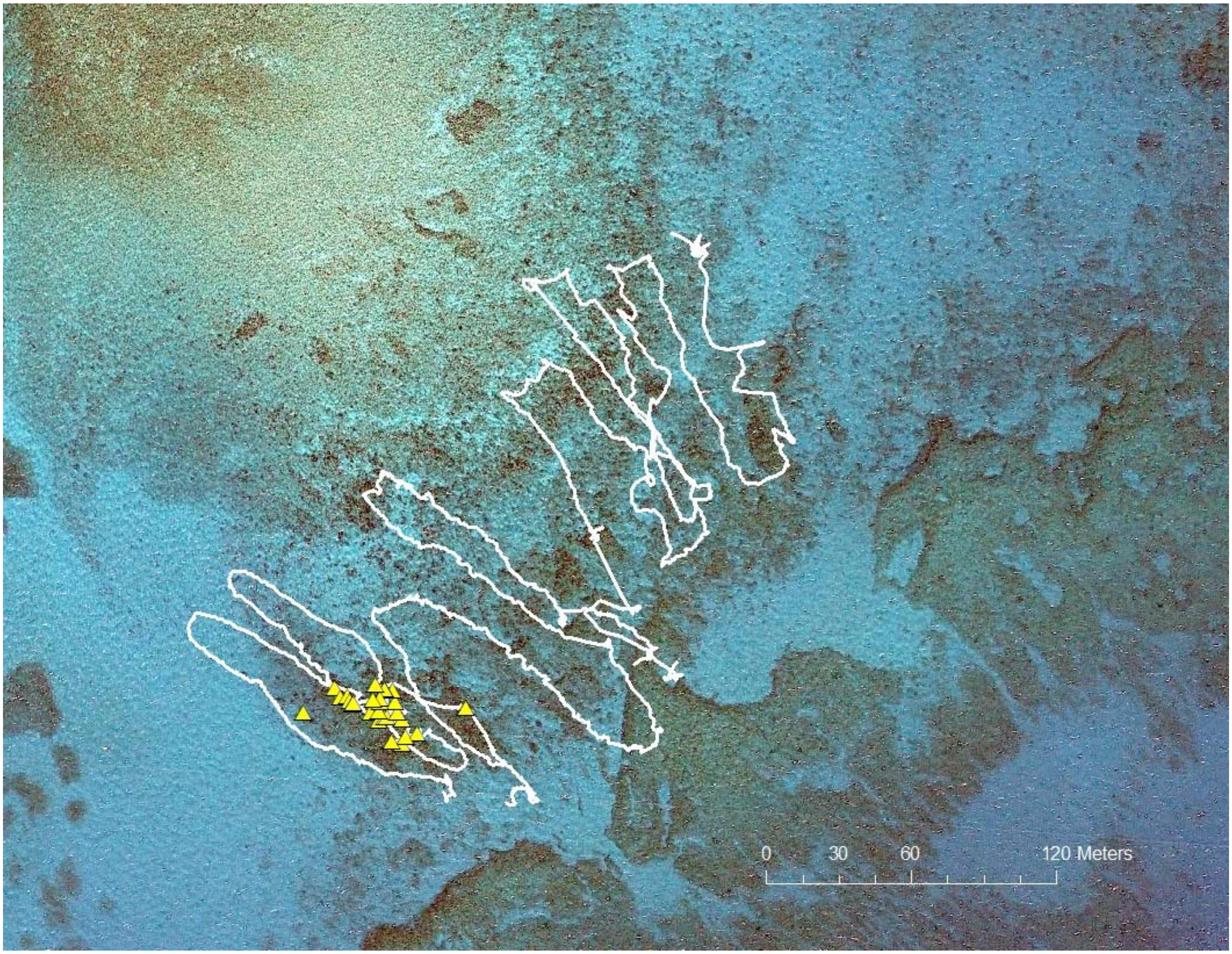


Sand Island 2015

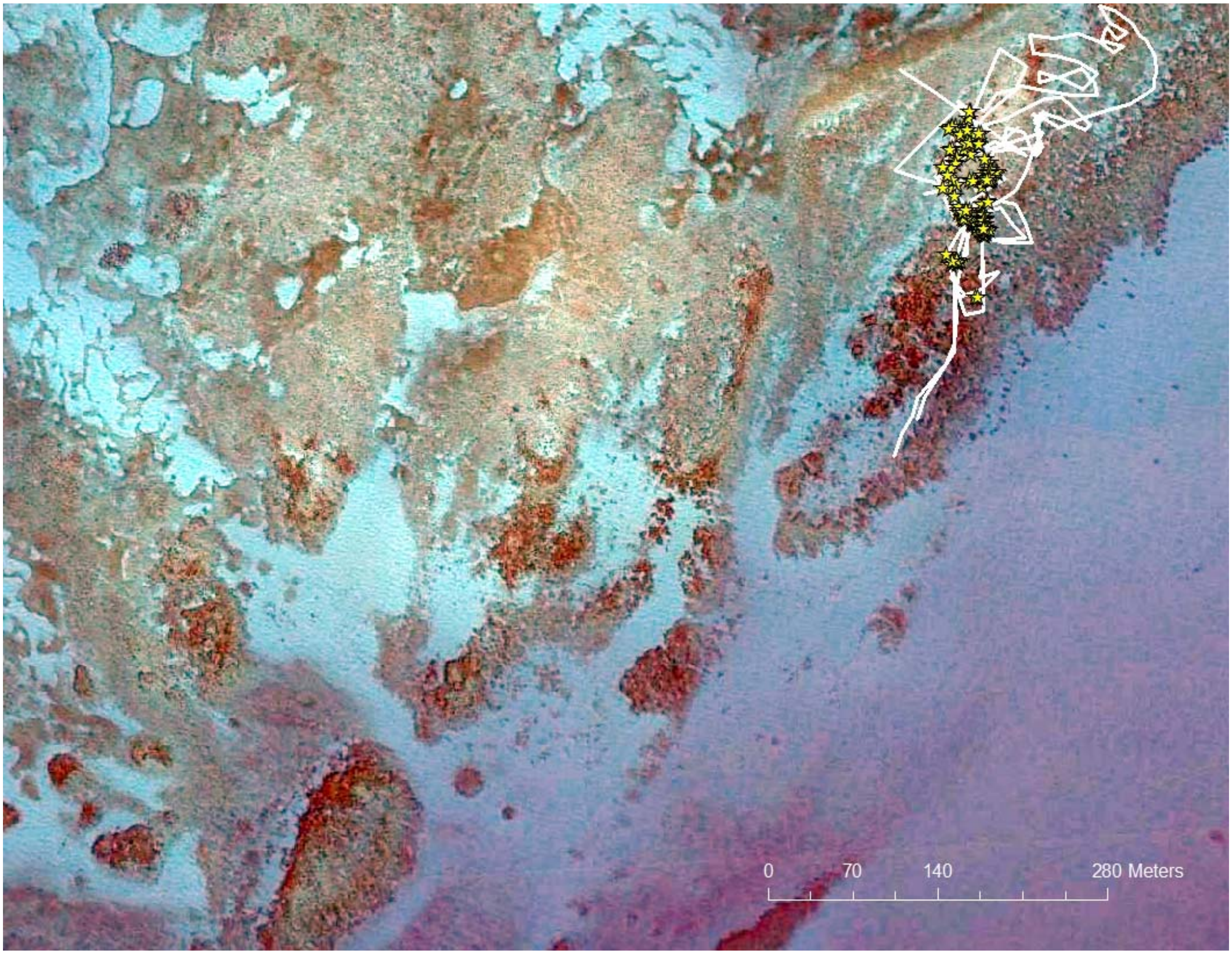


Watsons Reef 2006

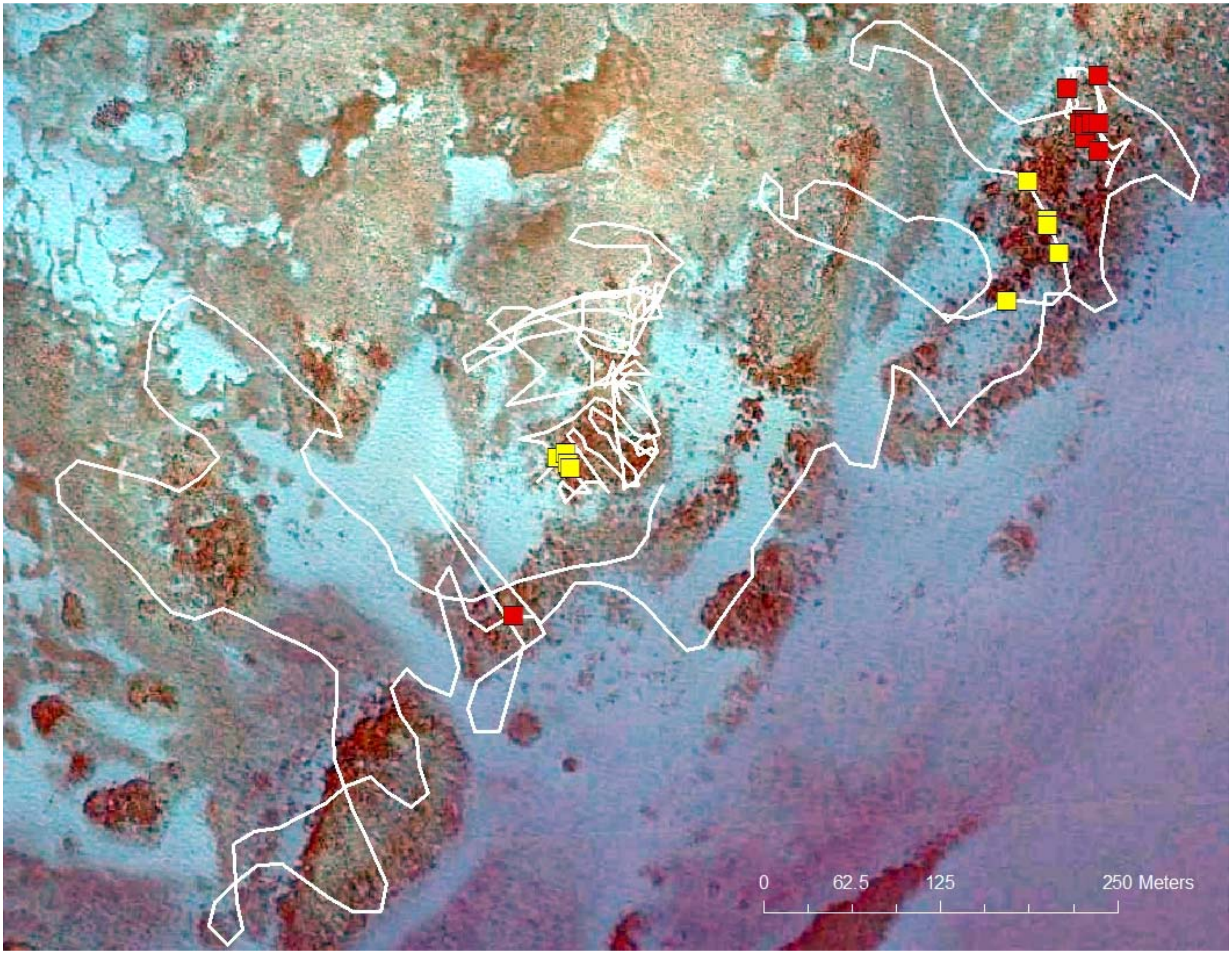


Watsons Reef 2007

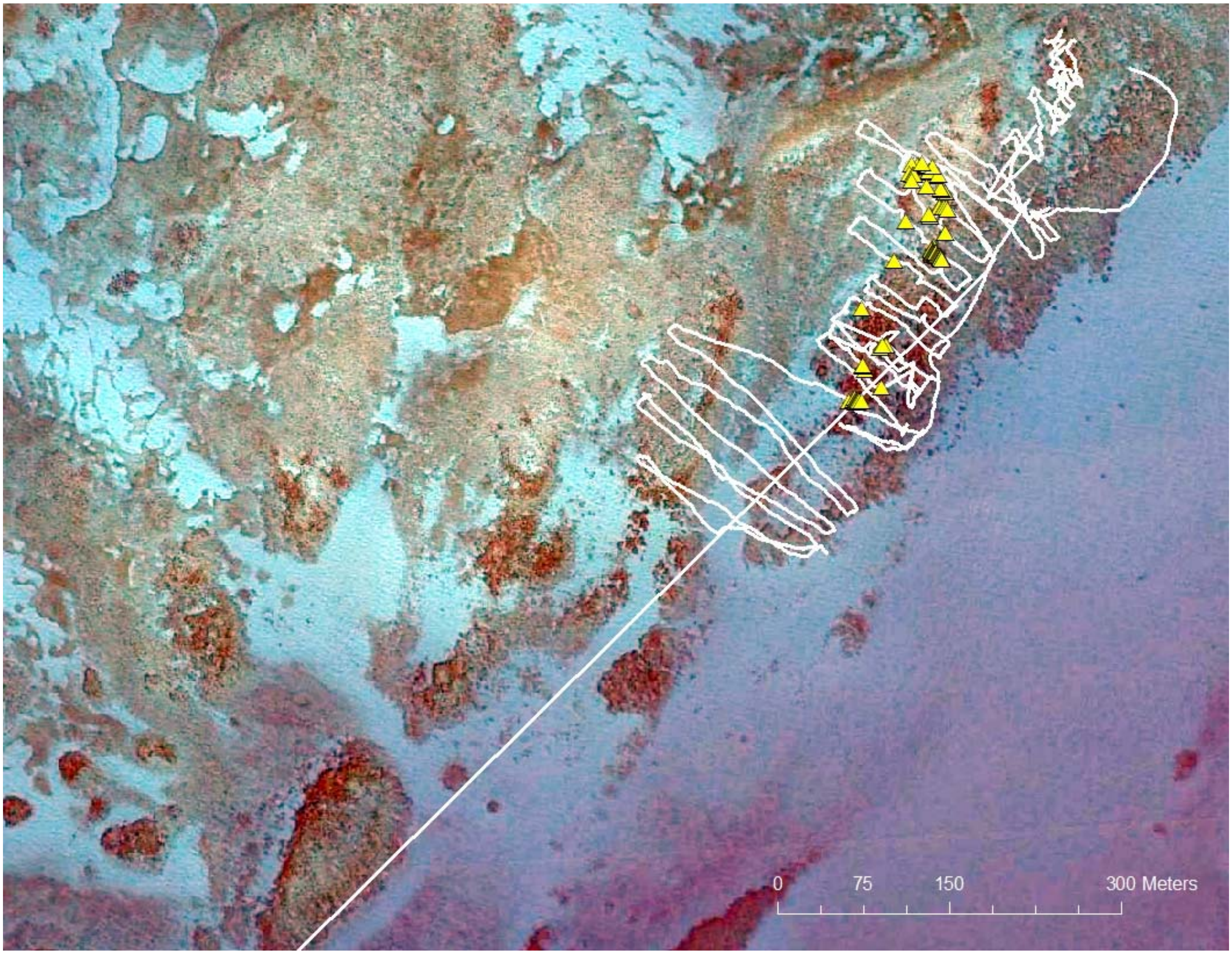


Watsons Reef 2015

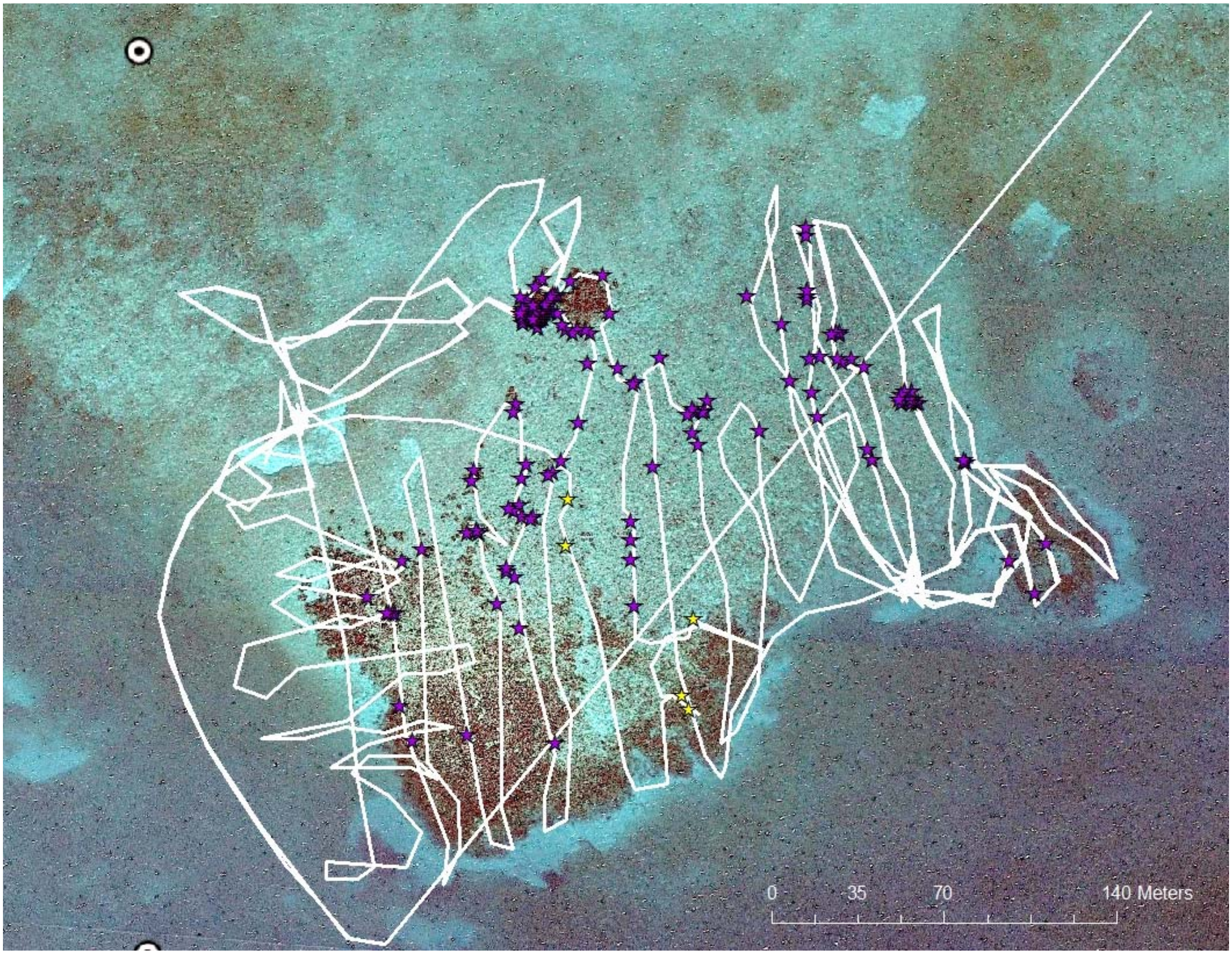


White Bank Dry Rocks #1 2006

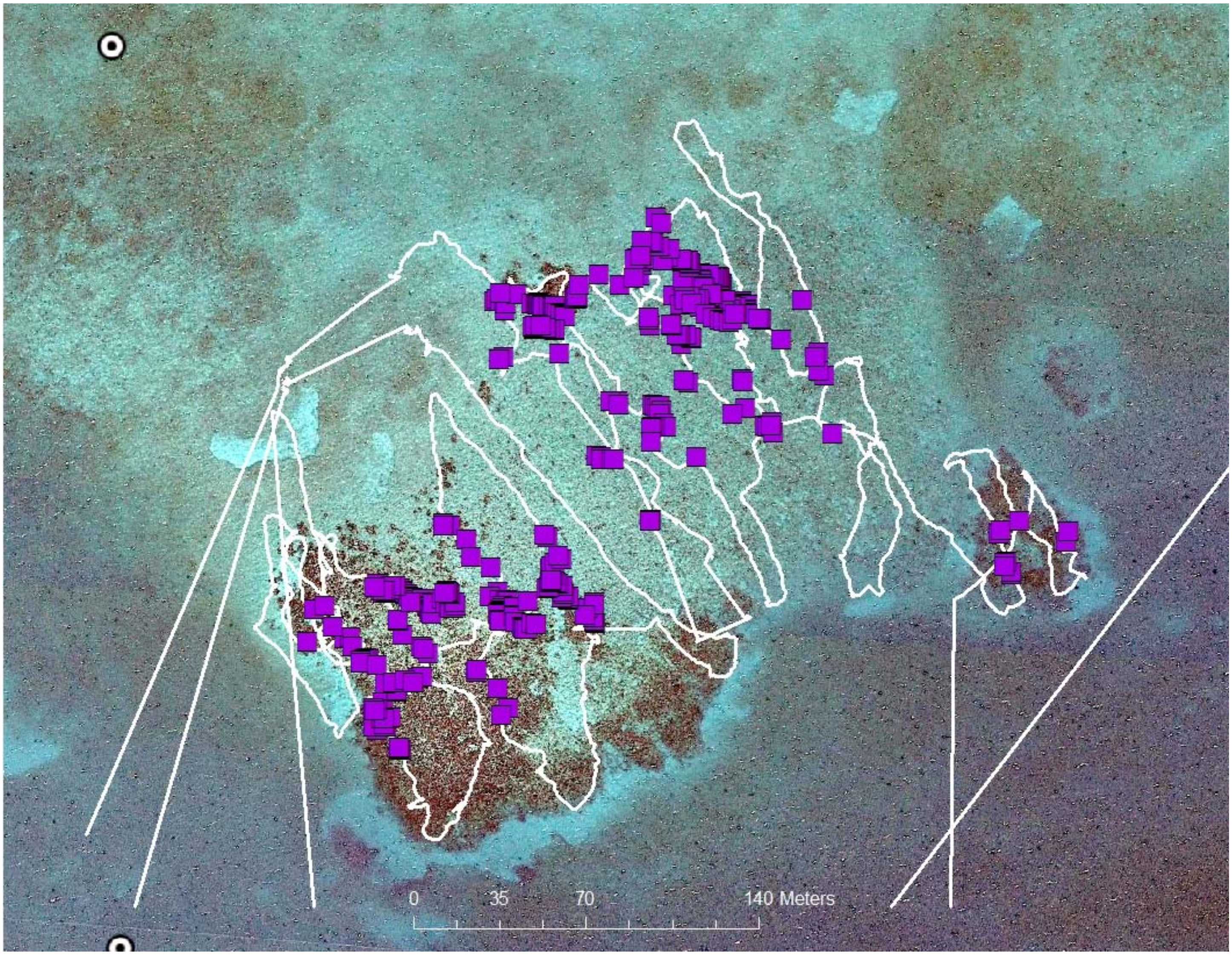


White Bank Dry Rocks #1 2014

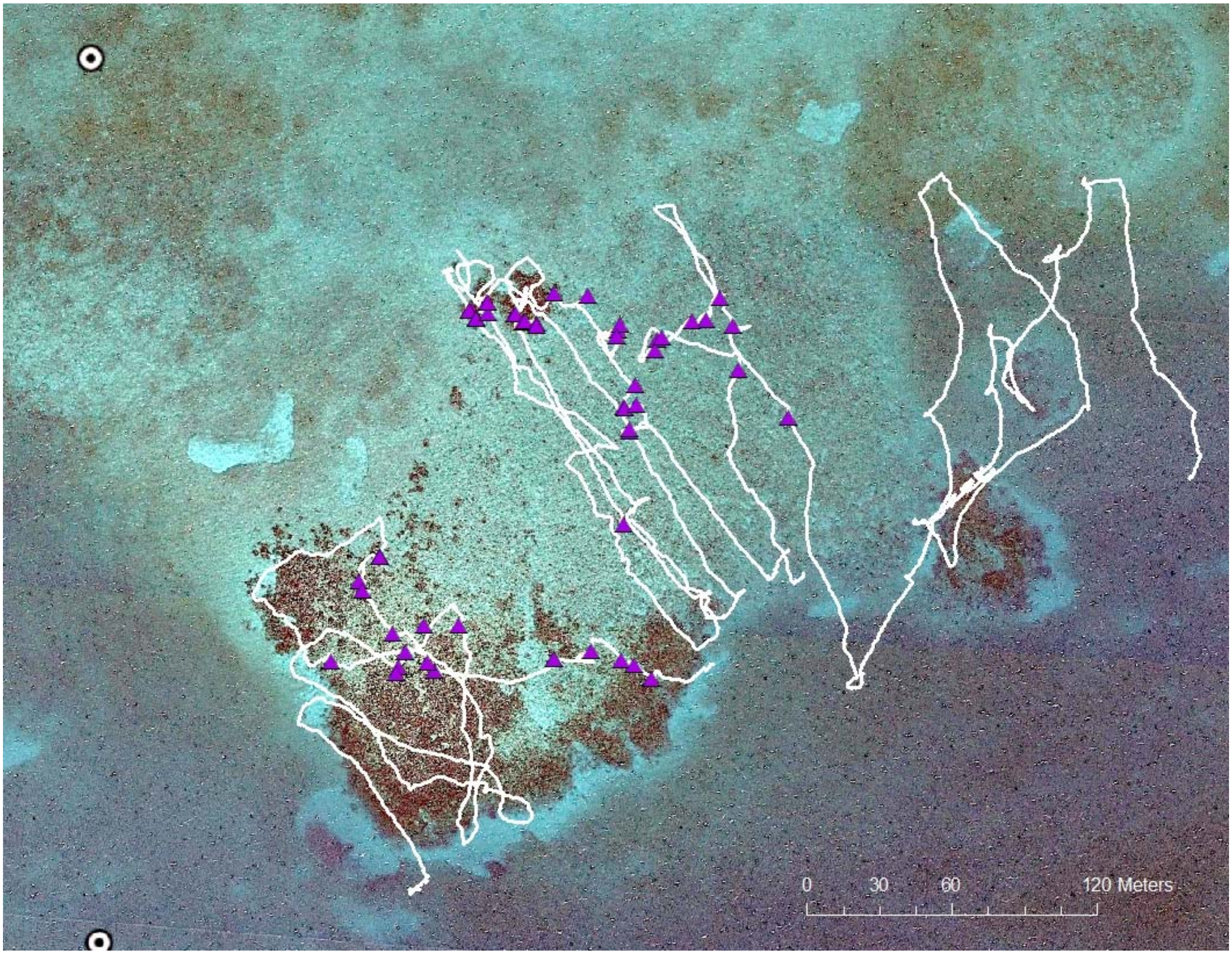


White Bank Dry Rocks #1 2015

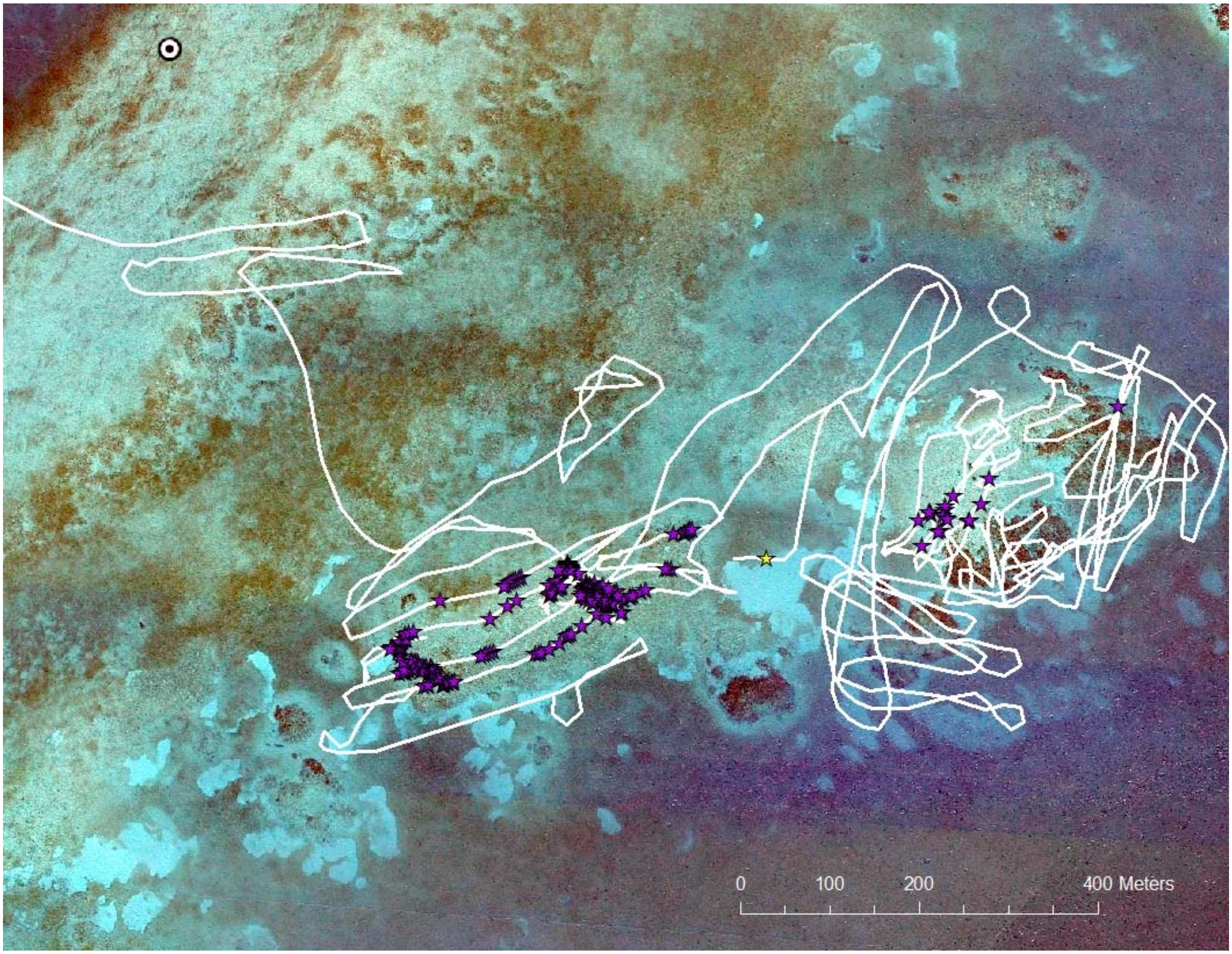


White Bank Dry Rocks #2 2006

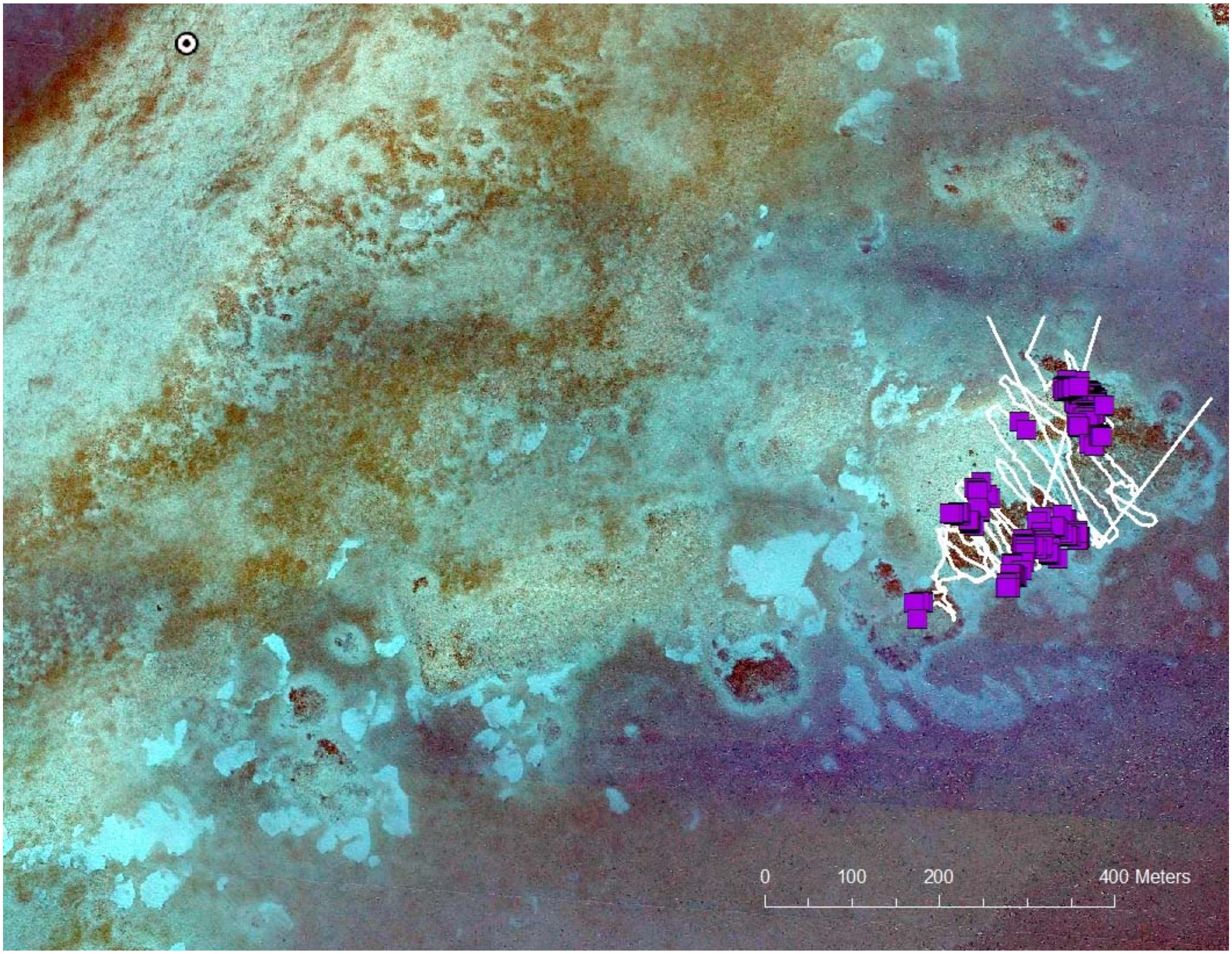


White Bank Dry Rocks #2 2014

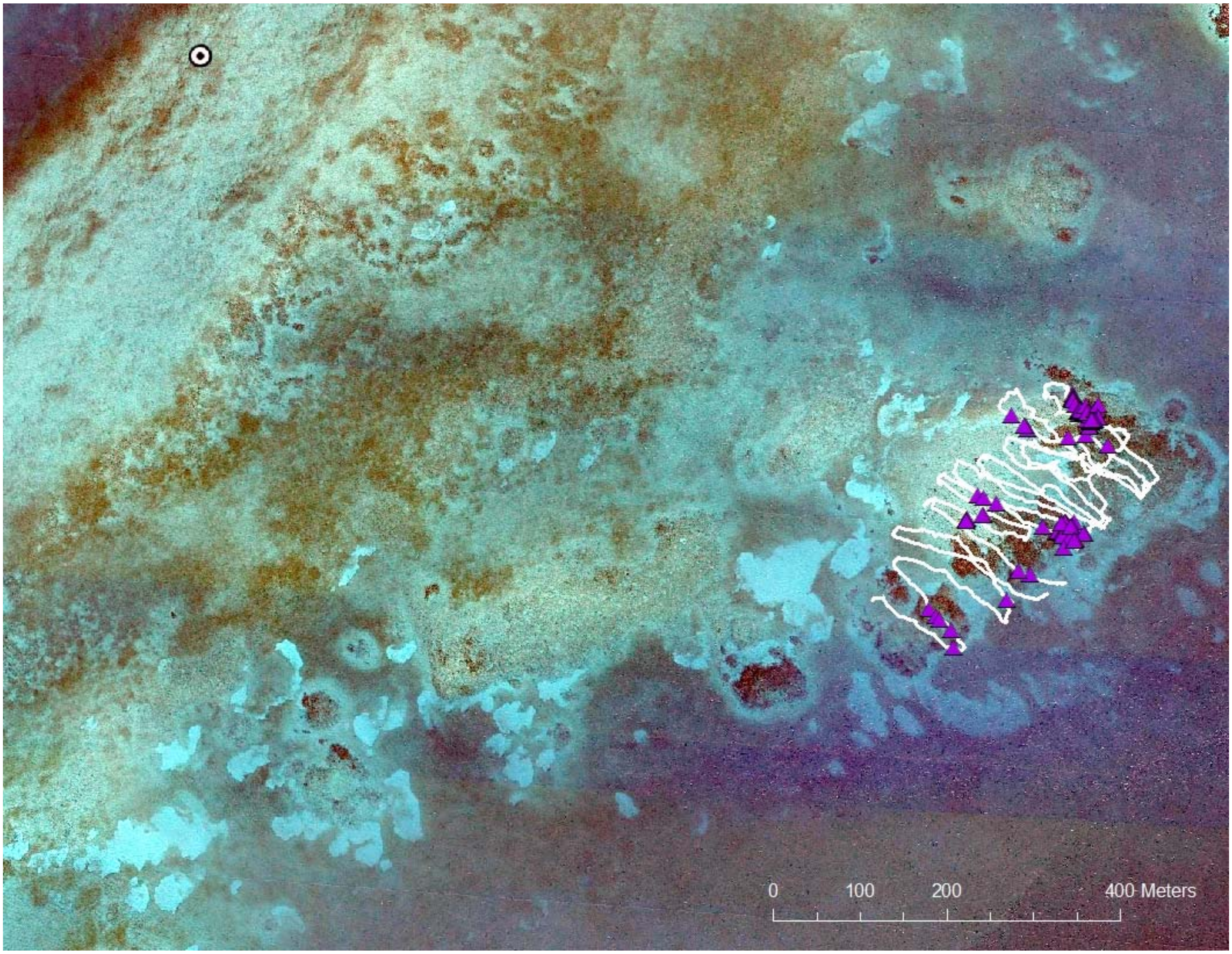


White Bank Dry Rocks #2 2015
