# Supplementary figures and images for: Reef-scale trends in Florida Acropora spp. abundance and the effects of population enhancement
Source: PeerJ. 2016 Sep 29;4:e2523. doi: 10.7717/peerj.2523 (PMC5047146; doi:10.7717/peerj.2523)

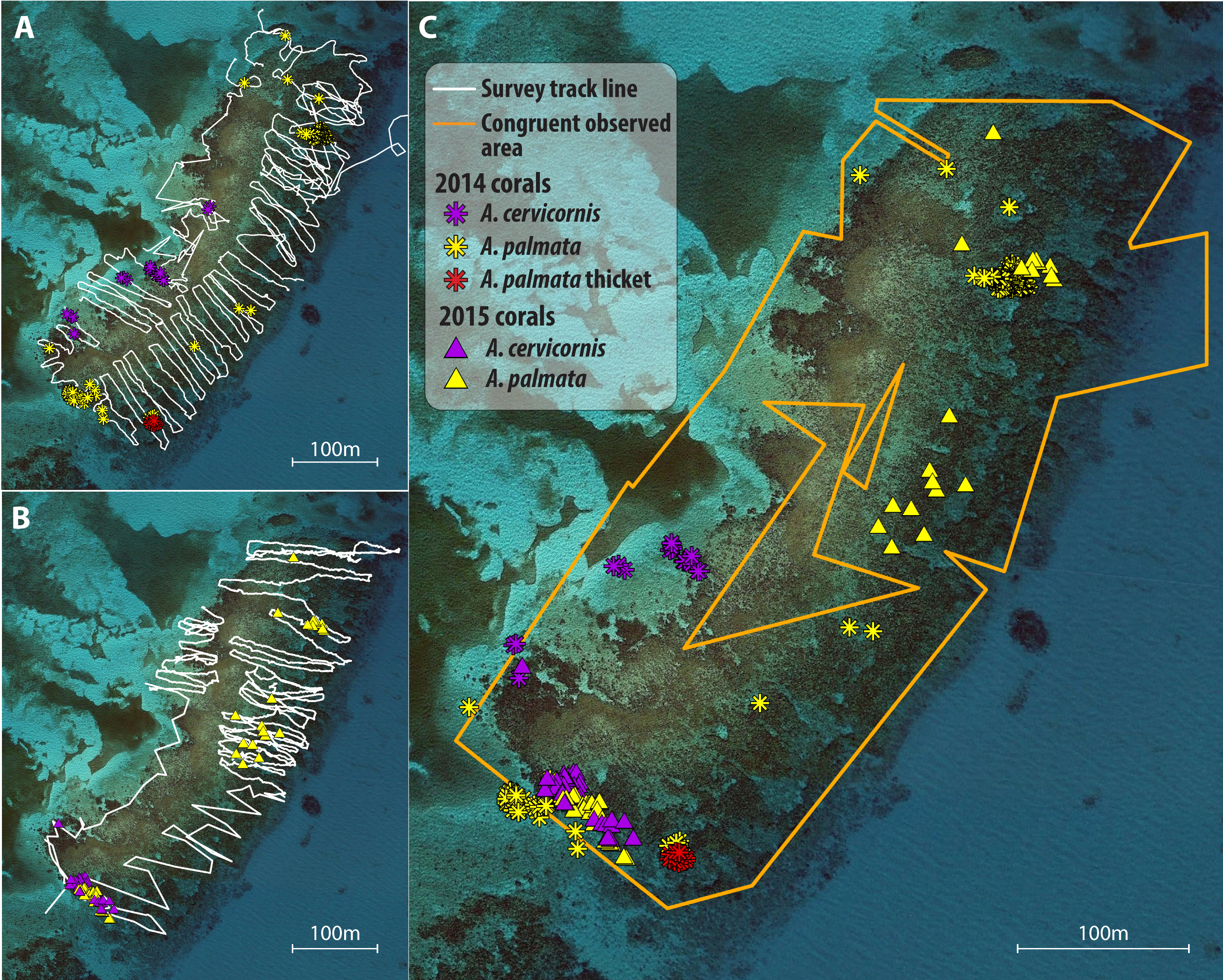

Supplement: Figure S2 — Observed search tracks and waypoint features mapped for Grecian Rocks reef between 2014 (A; waypoints as asterisks) and 2015 (B; waypoints as triangles). A. palmata colony waypoints are depicted in yellow, A. cervicornis colonies in purple, and A. palmata thicktet outline points in red. (C) Merged maps for temporal comparison showing the congruent observed area (determined by GIS intersect of the polygons determined by the search tracks for each year) for both years and the overlayed colony distribution observed in both years. [file peerj-04-2523-s006.png]
